# Vertebral Development in Paleozoic and Mesozoic Tetrapods Revealed by Paleohistological Data

**DOI:** 10.1371/journal.pone.0152586

**Published:** 2016-04-13

**Authors:** Marylène Danto, Florian Witzmann, Nadia B. Fröbisch

**Affiliations:** 1 Museum für Naturkunde, Leibniz-Institut für Evolutions- und Biodiversitätsforschung, Invalidenstraße 43, 10115 Berlin, Germany; 2 Department of Ecology and Evolutionary Biology, Brown University, Providence, Rhode Island, RI 02912, United States of America; College of the Holy Cross, UNITED STATES

## Abstract

Basal tetrapods display a wide spectrum of vertebral centrum morphologies that can be used to distinguish different tetrapod groups. The vertebral types range from multipartite centra in stem-tetrapods, temnospondyls, and seymouriamorphs up to monospondylous centra in lepospondyls and have been drawn upon for reconstructing major evolutionary trends in tetrapods that are now considered textbook knowledge. Two modes of vertebral formation have been postulated: the multipartite vertebrae formed first as cartilaginous elements with subsequent ossification. The monospondylous centrum, in contrast, was formed by direct ossification without a cartilaginous precursor. This study describes centrum morphogenesis in basal tetrapods for the first time, based on bone histology. Our results show that the intercentra of the investigated stem-tetrapods consist of a small band of periosteal bone and a dense network of endochondral bone. In stereospondyl temnospondyls, high amounts of calcified cartilage are preserved in the endochondral trabeculae. Notably, the periosteal region is thickened and highly vascularized in the plagiosaurid stereospondyls. Among “microsaur” lepospondyls, the thickened periosteal region is composed of compact bone and the notochordal canal is surrounded by large cell lacunae. In nectridean lepospondyls, the periosteal region has a spongy structure with large intertrabecular spaces, whereas the endochondral region has a highly cancellous structure. Our observations indicate that regardless of whether multipartite or monospondylous, the centra of basal tetrapods display first endochondral and subsequently periosteal ossification. A high interspecific variability is observed in growth rate, organization, and initiation of periosteal ossification. Moreover, vertebral development and structure reflect different lifestyles. The bottom-dwelling Plagiosauridae increase their skeletal mass by hyperplasy of the periosteal region. In nectrideans, the skeletal mass decreases, as the microstructure is spongy and lightly built. Additionally, we observed that vertebral structure is influenced by miniaturization in some groups. The phylogenetic information that can be drawn from vertebral development, however, is limited.

## Introduction

**Fig 1:**
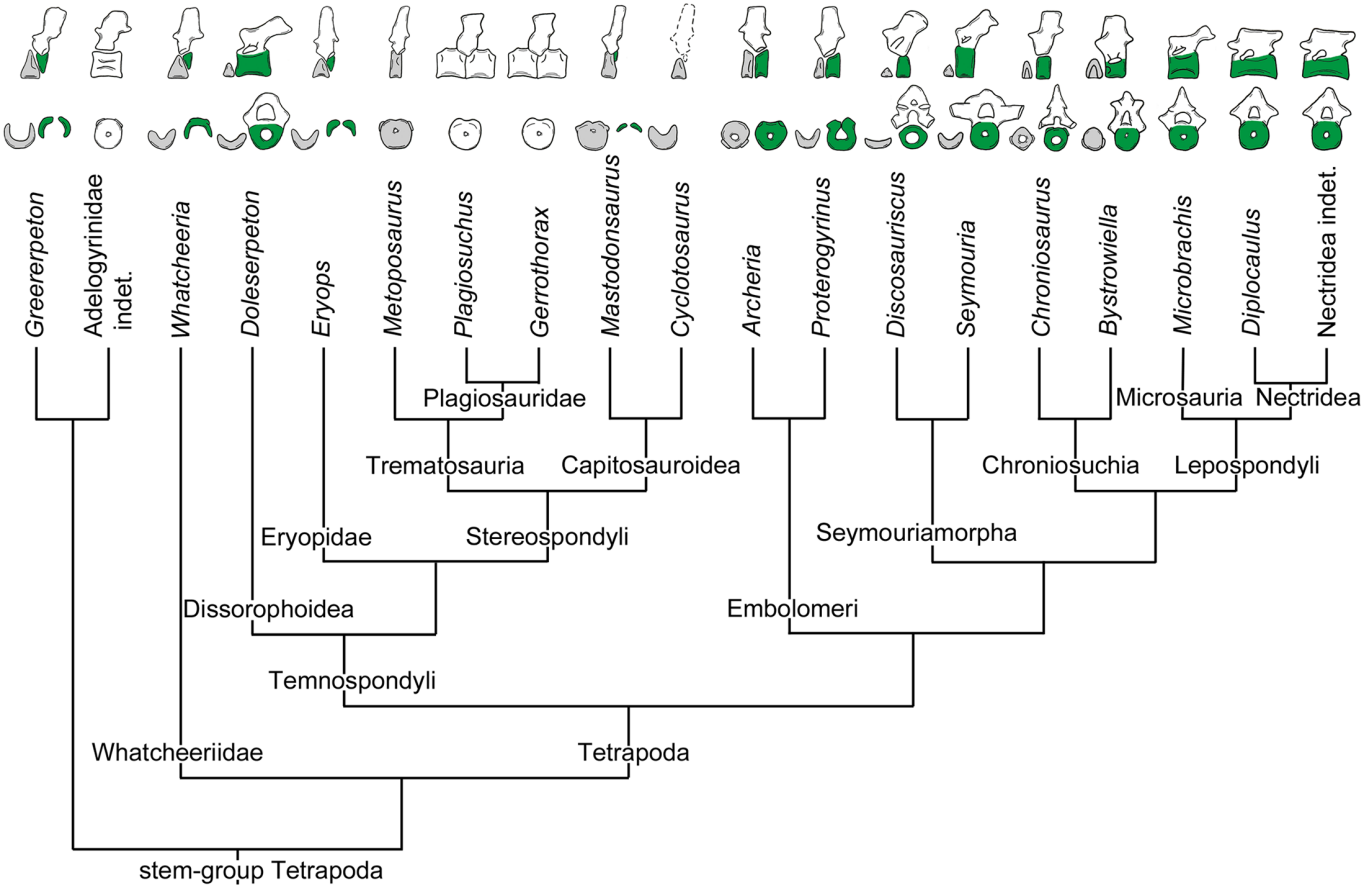
Phylogenetic relationship of extinct tetrapods examined in this study. After [4, 10, 65, 66]. The sizes of the vertebral elements are not to scale and have been redrawn from [16, 21, 24, 34, 41, 67–73]. Grey: intercentrum; green: pleurocentrum; white: origin unresolved.

Basal tetrapods occurred from the Carboniferous up to the Early Cretaceous [1,2]. In the present study, we follow the taxonomic definitions and phylogenetic framework of Ruta et al. [3] and Ruta & Coates [4]: hence, “basal tetrapods” or “crown-group Tetrapoda” encompasses the Paleozoic and Mesozoic temnospondyls, anthracosaurs, seymouriamorphs and lepospondyls. Falling outside and nested below the crown-group tetrapods are the tetrapod stem-groups, including Devonian taxa such as *Acanthostega* and more derived Carboniferous taxa, exemplified by *Whatcheeria* and *Greererpeton*. From fish ancestors they radiated into several different clades, adapting to aquatic, amphibious, and terrestrial habitats. Commonly accepted by most authors [3–6] but still controversial [7–9], basal tetrapods are subdivided into the lissamphibian stem-group (temnospondyls) and the amniote-stem group (anthracosaurs, chroniosuchia, seymouriamorphs, and lepospondyls) (Fig 1). Temnospondyls are a large and diverse group of Paleozoic and Mesozoic tetrapods [10]. They show a great diversity of body forms, ranging from miniaturized forms (around 5cm body length) up to large forms (around 5m body length) [11]. Embolomeres comprise crocodile-like tetrapods, which are grouped within the paraphyletic anthracosaurs [12, 13]. They range from small, terrestrial to larger, fully aquatic forms [14]. Chroniosuchians form a small clade of anthracosaur-like tetrapods and are mainly known from the Permian and Triassic of Russia and Germany [15, 16]. They are small to medium-sized varanid or crocodile-like animals and are characterized by the distinct morphology of their osteoderms and vertebral centra [17, 18]. Another small group of Paleozoic tetrapods are the seymouriamorphs found in Lower and Upper Permian localities in North America, Europe, and Asia [19–22]. The systematic position of this group is still debated [3, 23]. Seymouriamorphs have heavily ossified postcrania with robust limbs indicating a primarily terrestrial mode of life as adults [24]. Lepospondyls are a group of mostly small, but morphologically heterogeneous basal tetrapods. They range from the Early Carboniferous to the Early Permian [25] with recent phylogenetic analyses indicating that they are a monophyletic group [3, 4, 26].

Initially, all stem-tetrapods and basal tetrapods were grouped into one of two large groups either “labyrinthodonts” or lepospondyls [25, 27, 28]. “Labyrinthodonts” (i.e. temnospondyls, anthracosaurs, and seymouriamorphs) were a paraphyletic assemblage of mostly large tetrapods and were recognized by the labyrinthodont infolding of the teeth (shared with their fish-like relatives, [29]) and multipartite vertebral centra. One vertebral segment is composed of a neural arch, an unpaired intercentrum, and paired or unpaired pleurocentra. Lepospondyls, instead, were distinguished by their monospondylous, cylindrical vertebral centra, a small body size, and the absence of a labyrinthodont infolding of dentine and enamel. With the advance of cladistic analyses, most authors have rejected this subdivision, as it disregarded important morphological and biological differences (see [24] and references therein).

However, vertebral structures can still be used to distinguish the main tetrapod groups (Fig 1): Stem-tetrapods and most temnospondyls have the so-called rhachitomous vertebral construction where one vertebral segment is subdivided into three vertebral elements. The unpaired, crescentic intercentrum embraces the notochord from the ventral side and forms the main element of the vertebral body. The dorsal portion is composed of paired, small pleurocentra. The neural arch is supported by the pleurocentra and the intercentrum bears the parapophysis for articulation with the ribs [30]. As the rhachitomous vertebral type resembles that seen in tetrapodomorph fishes like *Eusthenopteron* [31] and *Panderichthys* [32], it is generally considered to be the ancestral condition for tetrapods [24, 33]. The stereospondylous vertebral type is characterized by a large, disk-shaped intercentrum. The paired pleurocentra are reduced in size and may also be absent or unossified [34, 35]. This vertebral pattern is characteristic for the Stereospondyli, a monophyletic group of diverse, mainly Triassic temnospondyls [10]. Notably, the Plagiosauridae among the Stereospondyli display a different vertebral construction. A vertebral unit is composed of a single, spool-shaped vertebral centrum and an intervertebral neural arch, i.e. the neural arch is situated between two adjacent centra [36]. The amniote-stem group (embolomeres, chroniosuchians, seymouriamorphs, and lepospondyls) have the so-called gastrocentrous vertebral type. In these groups, the pleurocentrum forms the dominant vertebral element, while the intercentrum is reduced in size or may be fully absent. In most embolomeres, both the inter- and pleurocentrum form fully ossified discs that are centrally pierced by the notochordal canal [24, 37]. A ball-and socket joint between vertebral centra can be found within the chroniosuchians [15, 16]. The spool-shaped, deeply amphicoelous pleurocentrum is fused to the neural arch, whereas the smaller intercentrum shows a ball-shaped morphology. In seymouriamorphs, the small intercentrum is crescent-shaped, whereas the cylindrical pleurocentrum is large and pierced by the notochordal canal. The neural arches are swollen and possess short neural spines [19, 22]. In contrast to these multipartite vertebral types, most lepospondyls possess only a single central element per segment, i.e. have monospondylous centra. This is generally regarded as the pleurocentrum and has a cylindrical shape and is often fused to the neural arch [30, 38–41]. In some “microsaur” groups, a small, wedge-shaped intercentrum is preserved [42–44]. This wide spectrum of vertebral structures in stem-tetrapods and basal tetrapods led different authors to deduce divergent phylogenetic trends and relationships [27, 33, 45, 46]. One example is the very influential scheme provided by Romer [47] in which he assumed that the vertebral body of amniotes can be traced back to the pleurocentrum of the rhachitomous vertebrae in tetrapodomorph fishes. Thus, the gastrocentrous vertebral type as seen in seymouriamorphs would lead to the amniote vertebra. Further examples are the monospondylous and spool-shaped vertebral centra of lepospondyls and lissamphibians. Gardiner [48] regarded the holospondylous morphology, and the presumably similar vertebral formation, as indication of a close phylogenetic relationship. This hypothesis is in accordance with cladistic analysis of Vallin & Laurin [7] and Marjanović & Laurin [23, 49].

Two modes of vertebral formation have been described among basal tetrapods [39, 44, 48, 50]: the multipartite vertebral centra of temnospondyls, anthracosaurs, and probably seymouriamorphs are ossified at a relatively late ontogenetic stage from cartilaginous precursors. In larvae and juveniles, only the neural arches are ossified and preserved as paired elements, and the vertebral centra appear subsequently as ill-defined ossifications. It was proposed that the vertebral centra formed first by chordal and subsequently by perichordal centrum formation [44]. Specifically, it was suggested that mesenchyme cells of the perichordal tube migrated into the notochordal sheath and chondrified there. The resulting chordal centrum then induced the development of the perichordal portion of the centrum. Thus, the vertebral centra grew from the inside out. In contrast, the monospondylous vertebral centra of lepospondyls ossified at a very early ontogenetic stage and approximately simultaneously with the neural arches. For instance, in a small specimen of the microsaur *Hyloplesion* (16mm length), the centra already form thin-walled cylinders [44]. It was hypothesized that the centra developed solely by the so called perichordal centrum formation in which the centra are derived from the mesenchyme cells of the perichordal tube and formed by direct ossification without a cartilaginous precursor [44]. The chordalcentrum stage was reduced or absent. In contrast to the multipartite vertebral centra, the monospondylous centra were thought to grow from the outside in [44]. However, at the time only few details of vertebral centra development in basal tetrapods were known because no histological investigations had been carried out.

Paleohistology of bones has become an important instrument in the investigation of life history in extinct vertebrates and can be used to determine different ossification processes [51, 52, 53]. Endochondral ossification, for example, is characterized by cartilaginous precursors that are substituted by bony tissue. In membranous ossification, however, bone is formed directly without a cartilaginous precursor. Similarly, periosteal bone ossifies directly and is formed by superposition of new bone layers on the bone surface. Most histological studies on basal tetrapods have focused on long bone histology in temnospondyls and seymouriamorphs (e.g. [54–58]). Until the present, histological investigations of vertebral centra were rare [59–64] and quite often represented by only short notes [59–62].

This study addresses the ontogenetic development of vertebral centra in Paleozoic and Mesozoic basal tetrapods based on their bone histology. It aims to classify which bone tissues compose the vertebral centra and how they vary between different groups of basal tetrapods. Specifically, the following questions are addressed: (1) in which basal tetrapods and in which parts of the centrum can evidence of endochondral ossification be found based on the bone structure? (2) is calcified cartilage preserved, and if so, in which taxa and in which ontogenetic stages? (3) in which basal tetrapods does the bone structure show evidence of membranous (i.e. direct) ossification? If membranous ossification is present, is the centrum solely formed by it (i.e., are cartilage and endochondral bone absent in the centrum)? And (4) to which extent is the vertebral formation influenced by the presumed lifestyle, and by overall body size of the respective taxon?

## Material and Methods

For the present study, vertebral centra of different basal tetrapod lineages were thin-sectioned (Table 1). The material belongs to the:

- Cleveland Museum of Natural History, Cleveland (CMNH)
- Department of Ecology, Bratislava (DE.KO)
- Field Museum of Natural History, Chicago (FMNH)
- Museum für Naturkunde Berlin (MB)
- National Museum of Scotland, Edinburgh (NMS)
- Paleontological Institute of the Russian Academy of Sciences, Moscow (PIN)
- Sam Noble Oklahoma Museum of Natural History (OMNH)
- Slovak National Museum, Bratislava (Z)
- Staatliches Museum für Naturkunde, Stuttgart (SMNS)

**Table 1:** List of specimens examined in this study.

**STEM-TETRAPODA**					
**taxon**	**vertebral element/ position**	**estimated ontogen. stage**	**slice number**	**cutting plane**	**stratigraphy/locality**
*Greererpeton* sp. (CMNH 1969–4)	intercentrum (presacral)	adult	1969.4-1G	transversal	Early Carboniferous/ Greer, West Virginia, USA
-"-	-"-	-"-	1969.4-2G	transversal	-"-
Adelogyrinidae indet. (NMS 1993.56.111)	centrum (presacral)	adult	1993–1	transversal	Carboniferous, Dora Bone Bed/ Cowdenbeath, Scotland
-"-	-"-	-"-	1993–2	transversal	-"-
-"-	-"-	-"-	1993–3	longitudinal	-"-
-"-	-"-	-"-	1993–4	longitudinal	-"-
*Whatcheeria deltae* (FMNH PR 3347)	intercentrum (presacral)	adult	3347–2	transversal	Early Carboniferous/ Keokuk Country, Iowa, USA
**TEMNOSPONDYLI**					
**taxon**	**vertebral element/ position**	**estimated ontogen. stage**	**slice number**	**cutting plane**	**stratigraphy/locality**
*Doleserpeton* sp. (OMNH 73530B)	pleurocentrum (indet.)	adult	73530–2	transversal	Permian/ Richards Spur, Oklahoma, USA
*Doleserpeton* sp. (OMNH 73530D)	pleurocentrum (indet.)	adult	73530–4	transversal	Permian/ Richards Spur, Oklahoma, USA
*Doleserpeton* sp. (OMNH 73530E)	pleurocentrum (indet.)	adult	73530–5	transversal	Permian/ Richards Spur, Oklahoma, USA
*Doleserpeton* sp. (OMNH 73530F)	pleurocentrum (indet.)	adult	73530–6	longitudinal	Permian/ Richards Spur, Oklahoma, USA
*Eryops* sp. (MB.Am.1529)	intercentrum (presacral)	adult	1529–1	transversal	Permian, Connar Ranch Bonebed/ Texas, USA
-"-	-"-	-"-	1529–2	longitudinal	-"-
*Metoposaurus* sp. (MB.Am. 1437.2)	intercentrum (presacral)	indet.	1437.2–1	transversal	Triassic, Keuper/ Opole Region, Poland
-"-	-"-	-"-	1437.2–2	longitudinal	-"-
*Plagiosuchus* sp. (SMNS 52266A)	centrum (indet.)	indet.	52266–1	transversal	Triassic, Lower Keupen/ Vellberg, Germany
-"-	-"-	-"-	52266–2	longitudinal	-"-
*Plagiosuchus* sp. (SMNS 52266B)	centrum (indet.)	indet.	52266–3	transversal	Triassic, Lower Keupen/ Vellberg, Germany
-"-	-"-	-"-	52266–4	longitudinal	-"-
*Plagiosuchus* sp. (SMNS 52266C)	centrum (indet.)	indet.	52266–5	transversal	Triassic, Lower Keupen/ Vellberg, Germany
-"-	-"-	-"-	52266–6	longitudinal	-"-
*Gerrothorax* sp. (SMNS 83451B)	centrum (indet.)	indet.	83451–3	transversal	Triassic, Ob. Lettenkeuper/ Germany
-"-	-"-	-"-	83451–4	longitudinal	-"-
*Mastodonsaurus* sp. (SMNS 84213A)	intercentrum (indet.)	indet.	84213–1	transversal	Triassic, Lower Keupen/ Vellberg, Germany
-"-	-"-	-"-	84213–2	longitudinal	-"-
*Cyclotosaurus intermedius* (MB.Am.1437.11)	intercentrum (indet.)	indet.	1437.11–1	transversal	Triassic/ Opole Region, Poland
-"-	-"-	-"-	1437.11–2	longitudinal	-"-
**EMBOLOMERI**					
**taxon**	**vertebral element/ position**	**estimated ontogen. stage**	**slice number**	**cutting plane**	**stratigraphy/locality**
*Archeria* sp. (MB.Am.1533)	- (indet.)	adult	1533–1	transversal	Permian/ Briar Creek, Texas, USA
*Proterogyrinus* sp. (CMNH 1969–4)	pleurocentrum (indet.)	adult	1969.4-1P	transversal	Early Carboniferous/ Greer, West Virginia, USA
**SEYMOURIAMORPHA**					
**taxon**	**vertebral element/ position**	**estimated ontogen. stage**	**slice number**	**cutting plane**	**stratigraphy/locality**
*Discosauriscus* sp. (DE.KO.187)	pleurocentrum (indet.)	juvenile	KO187-1	transversal	Lower Permian/ Kochov-Les, Czech Republic
*Discosauriscus austriacus* (Z15568 [K52])	pleurocentrum (indet.)	juvenile	K52-1	transversal	Lower Permian/ Kochov-Horka, Czech Republic
*Seymouria* sp. (OMNH 73499)	pleurocentrum (presacral)	adult	73499	transversal	Permian/ Richards Spur, Oklahoma, USA
**CHRONIOSUCHIA**					
**taxon**	**vertebral element/ position**	**estimated ontogen. stage**	**slice number**	**cutting plane**	**stratigraphy/locality**
*Chroniosaurus dongusensis* (PIN 3585–220)	pleurocentrum (indet.)	juvenile	3585.220	transversal	Permian, Donguz-6/ Orenburg region, Russia
*Chroniosaurus dongusensis* (PIN 3585–214)	intercentrum (caudal)	juvenile	3585.214	transversal	Permian, Donguz-6/ Orenburg region, Russia
*Chroniosaurus dongusensis* (PIN 3585–218)	intercentrum (presacral)	juvenile	3585.218	transversal	Permian, Donguz-6/ Orenburg region, Russia
*Bystrowiella schumanni* (SMNS 81872A)	pleurocentrum (indet.)	adult	81872–1	transversal	Triassic, Lower Keuper/ Herdtlingshagen, Germany
-"-	-"-	-"-	81872–2	longitudinal	-"-
*Bystrowiella schumanni* (SMNS 81872E)	intercentrum (presacral)	adult	81872–6	transversal	Triassic, Lower Keuper/ Herdtlingshagen, Germany
**LEPOSPONDYLI**					
**taxon**	**vertebral element/ position**	**estimated ontogen. stage**	**slice number**	**cutting plane**	**stratigraphy/locality**
Nectridea indet. (FMNH UR 2511)	pleurocentrum (presacral)	adult	2511	transversal	Early Permian/ Logan Country, Oklahoma, USA
*Diplocaulus magnicornis* (FMNH UR 2510)	pleurocentrum (caudal)	adult	2510	longitudinal	Early Permian, Arroyo Formation/ Wilbarger Country, Texas, USA
*Microbrachis* sp. (MB.Am.854)	pleurocentrum (indet.)	indet.	854–1	transversal	Carboniferous, Westphalian D/ Nýřany, Czech Republic
*Microbrachis* sp. (MB.Am.865)	pleurocentrum (indet.)	indet.	865	longitudinal	Carboniferous, Westphalian D/ Nýřany, Czech Republic

In most cases, the vertebral centra studied were completely preserved. The determination of a precise ontogenetic stage of a vertebral centrum remains difficult for several reasons. First, the majority of vertebrae used for thin sectioning are isolated and disarticulated material. Second, the size of a vertebral centrum depends on the age of a specimen, on its individual growth rate, and on the position of the bone along the vertebral column. The use of skeletochronological features such as growth marks for age determination is not reliable either, as the compact external cortex is often largely destroyed or the bone has been resorbed and remodeled [74]. In this study, 29 vertebral centra (intercentra and pleurocentra) of 20 taxa were examined.

The vertebral centra were embedded in synthetic resin and subsequently cut with a standard wet saw either along the transversal or/and longitudinal axis. The samples were then polished to a thickness of approximately 30–50μm and examined under a Zeiss Axioskop 40 microscope using transmitted ordinary and polarized light in magnifications ranging from 25x to 200x. Images were taken with an AxioCam MRc5 (Zeiss) camera. In addition, all sections were scanned with the Epson Perfection V600 Photo Scan. Histological terminology follows Francillon-Vieillot et al. [51].

## Results

### General Microanatomy and histology

To obtain an overview of the developmental origin of the vertebral centra, bones were thin-sectioned both along transversal and longitudinal planes (Table 1). In general, all the examined inter- and pleurocentra display a similar microstructural organization: the periosteal bone forms the ventro-lateral border of the vertebrae and the dorsally-located endochondral region forms a disorganized trabecular network. We observed interspecific variation in the size and structure of the periosteal region, the amount of calcified cartilage within the endochondral region, and the arrangement of bone around the notochordal canal.

### *Greererpeton* sp. (CMNH 1969.4-1G)–Stem-tetrapod, Colosteidae

**Fig 2:**
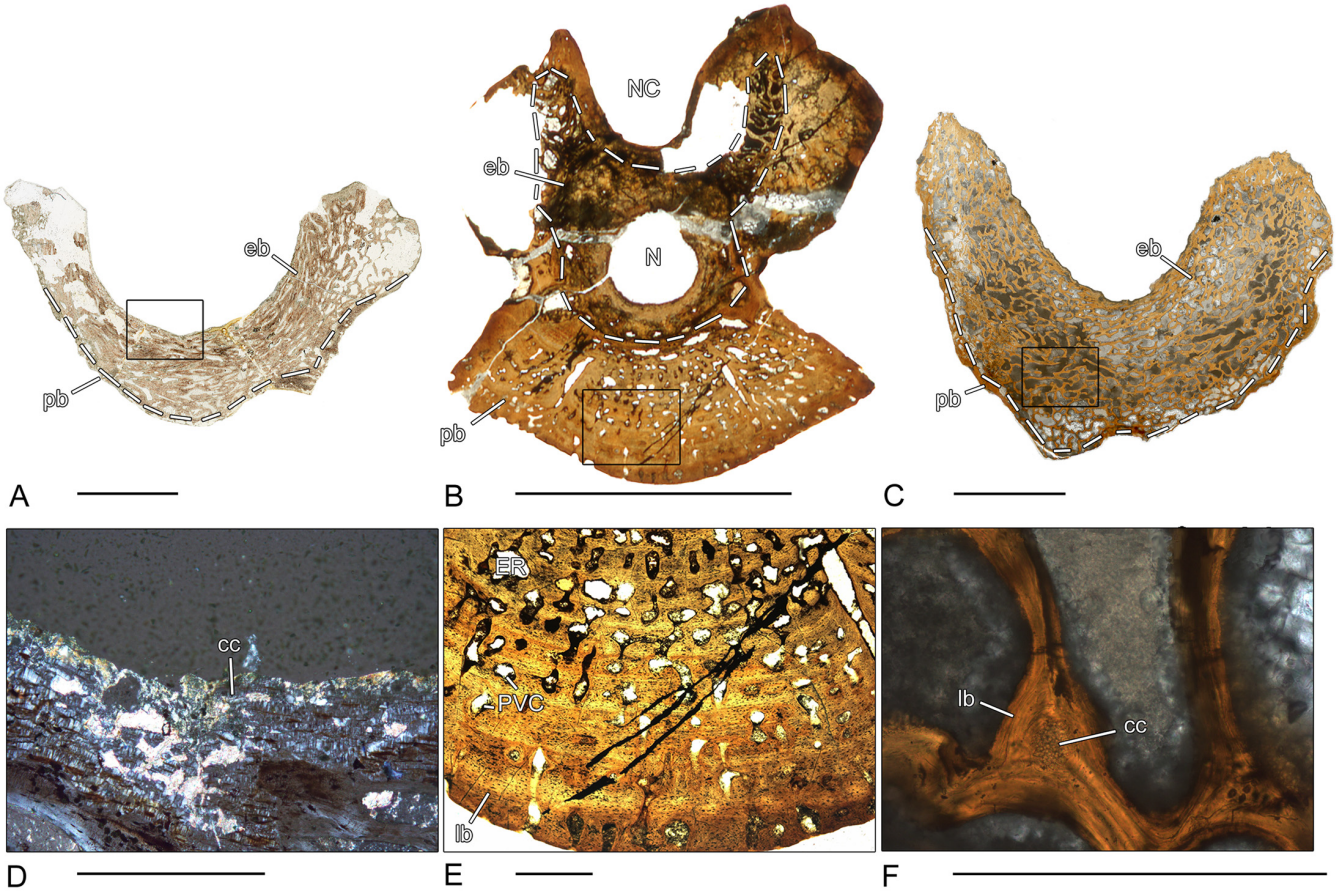
Vertebral histology of *Greererpeton* sp. (Stem-tetrapod, Colosteidae), Adelogyrinidae indet. (Stem-tetrapod, Adelospondyli), and *Whatcheeria deltae* (Stem-tetrapod, Whatcheeriidae). (A) Transverse section (1969.4-1G) of an intercentrum of *Greererpeton* sp. (CMNH 1969–4). (B) Transverse section (1993–2) of a centrum of Adelogyrinidae indet. (NMS 1993.56.111). (C) Transverse section (3347–2) of an intercentrum of *Whatcheeria deltae* (FMNH PR 3347). (D) Close-up of (A), in polarized light; calcified cartilage covers the dorsal surface of the intercentrum. (E) Close-up of (B), in normal transmitted light; thickened, spongy periosteal region with simple vascular canals and erosion rooms. (F) Close-up of (C), in normal transmitted light; remodeled trabecula that contains a core of primary calcified cartilage. Dotted line in (A), (B), and (C) indicates the border between periosteal and endochondral bone. In all centra, dorsal is to the top. Scale bars in (A)-(C) equal 5mm, in (D)-(F) 500μm. Abbreviations: cc—calcified cartilage; eb—endochondral bone; ER—erosion room; lb—lamellar bone; N—notochordal canal; NC—neural canal; pb—periosteal bone; PVC—primary vascular canal.

Unfortunately, the microanatomy of the crescentic intercentrum of *Greererpeton* is only incompletely preserved (Fig 2A). The thin, compact, periosteal bone forms the ventro-lateral border and is composed of lamellar bone. Internally, the structure of the periosteal bone becomes spongier. The periosteal bone is permeated by numerous short Sharpey’s fibers. The cancellous endochondral bone forms a dense and compact network of mostly remodeled trabeculae. Remnants of calcified cartilage are absent in the endochondral bone. Both the dorsal surface as well as the dorsal peaks are covered by calcified cartilage (Fig 2D).

### Adelogyrinidae indet. (NMS 1993–2)–Stem-tetrapod, Adelospondyli

The periosteal region is thickened with a slightly spongy structure and forms the major part of the spool-shaped centrum (Fig 2B and 2E). Dorsally, it is nearly avascular and covers the floor of the neural canal. Most of the vascular canals are simple vascular canals but the number of primary and secondary osteons increases towards the center. A few larger erosion rooms are also discernible. Sharpey’s fibers cannot be found. The endochondral region is situated dorsally and forms a dense network of parallel-fibered trabeculae, but is only incompletely preserved. The centrally located, rounded notochordal canal is surrounded by nearly avascular, compact bone.

### *Whatcheeria deltae* (FMNH PR 3347–2)–Stem-tetrapod, Whatcheeriidae

A very thin periosteal region forms the ventrolateral border of the crescentic intercentrum (Fig 2C and 2F). This lamellar bone is pierced by vascular canals and bays of erosions. Sharpey’s fibers cannot be found. The cancellous network of endochondral bone forms the major part of the centrum. The thin trabeculae display various stages of resorption and remodeling. In the dorsal peaks, the amount of calcified cartilage is high but decreases ventrally. The dorsal surface of the intercentrum is partly covered by a thin layer of calcified cartilage.

### *Doleserpeton* sp. (OMNH 73530–2; 73530–4; 73530–5; 73530–6)–Temnospondyli, Dissorophoidea

**Fig 3:**
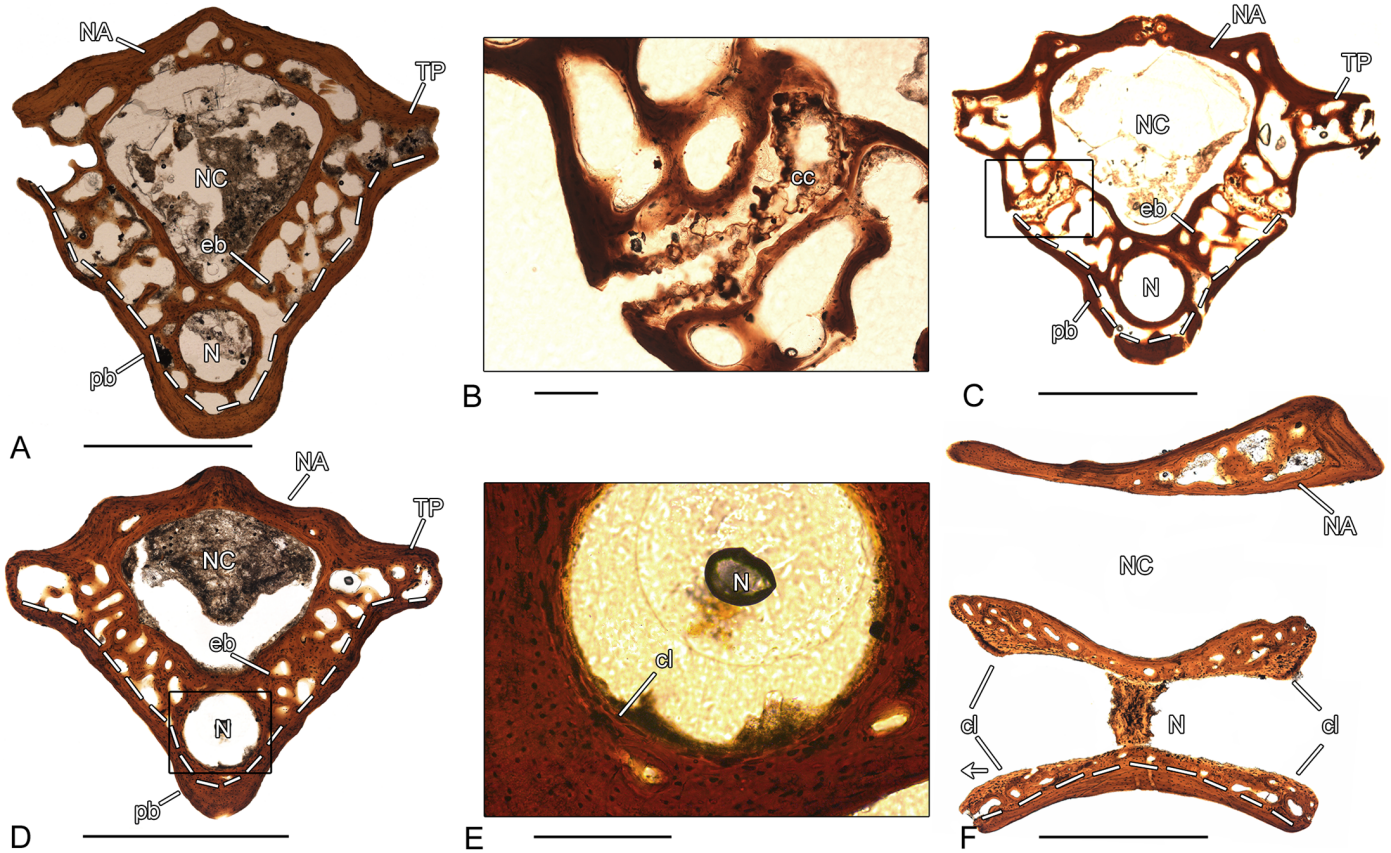
Vertebral histology of *Doleserpeton* sp. (Temnospondyli, Dissorophoidea). (A) Transverse section (73530–2) of a pleurocentrum of *Doleserpeton* sp. (OMNH 73530B). (B) Close-up of (C), in normal transmitted light; suture between pleurocentrum and neural arch with remnants of calcified cartilage. (C) Transverse section (73530–5) of a pleurocentrum of *Doleserpeton* sp. (OMNH 73530E). (D) Transverse section (73530–4) of a pleurocentrum of *Doleserpeton* sp. (OMNH 73530D). (E) Close-up of (D), in normal transmitted light; notochordal canal surrounded by large cell lacunae. (F) Longitudinal section (73530–6) of a pleurocentrum of *Doleserpeton* sp. (OMNH 73530F). Dotted line in (A), (C), (D), and (F) indicates the border between periosteal and endochondral bone. In all centra, dorsal is to the top. In (F), the arrowhead points anteriorly. Scale bars in (A), (C), (D), and (F) equal 1mm, in (B) and (E) 100μm. Abbreviations: cc—calcified cartilage; cl—cell lacuna; eb—endochondral bone; N—notochordal canal; NA—neural arch; NC—neural canal; pb—periosteal bone; TP—transverse process.

The three spool-shaped pleurocentra 73530–2, 73530–4 and 73530–5 display slight differences in their microstructural organization. In all investigated vertebral centra, the pleurocentrum and neural arch are fused, independent of their size. Only in the specimen 73530–5 (Fig 3C), a suture with remnants of cartilage is visible between centrum and neural arch (Fig 3D). The periosteal bone has a compact, avascular structure and consists of lamellar bone. It forms the ventrolateral border of the pleurocentrum and extents dorsally up to the transverse processes. The dorso-centrally located endochondral region is composed of broad remodeled trabeculae. In 73530–2, the trabeculae form a loose network with large intertrabecular spaces (Fig 3A). By contrast, the endochondral region is distinctly more compact in 73530–4 (Fig 3D). Calcified cartilage is absent in the endochondral bone and the notochordal canal is large and centrally located. In 73530–2 and 73530–5, the canal is surrounded by a ring of compact periosteal bone with an internal layer made up of large cell lacunae. In contrast, in pleurocentrum 73530–4, the notochordal canal is completely surrounded by endochondral and periosteal bone (Fig 3D). A distinct bony ring as in 73530–2 and 73530–5 is not recognizable. Large cell lacunae are still visible on the inner border (Fig 3E). The neural arch extends dorsally around the large neural canal and consists of lamellar bone with a compact and nearly avascular structure. In 73530–5, the neural arch consists of two lateral halves that are not yet fused. One pleurocentrum (73530–6) was sectioned longitudinally (Fig 3F). The ventral side is made up of compact, lamellar periosteal bone.. The concave (amphicoelous) articular surfaces of the centrum are characterized by an area of large cell lacunae. The neural arch is separated from the centrum by the large neural canal. The compact bone structure of the anterior part of the neural arch progresses posteriorly to a more spongy bone structure with extended primary bone deposits.

### *Eryops* sp. (MB.Am. 1529–1)–Temnospondyli, Eryopoidae

**Fig 4:**
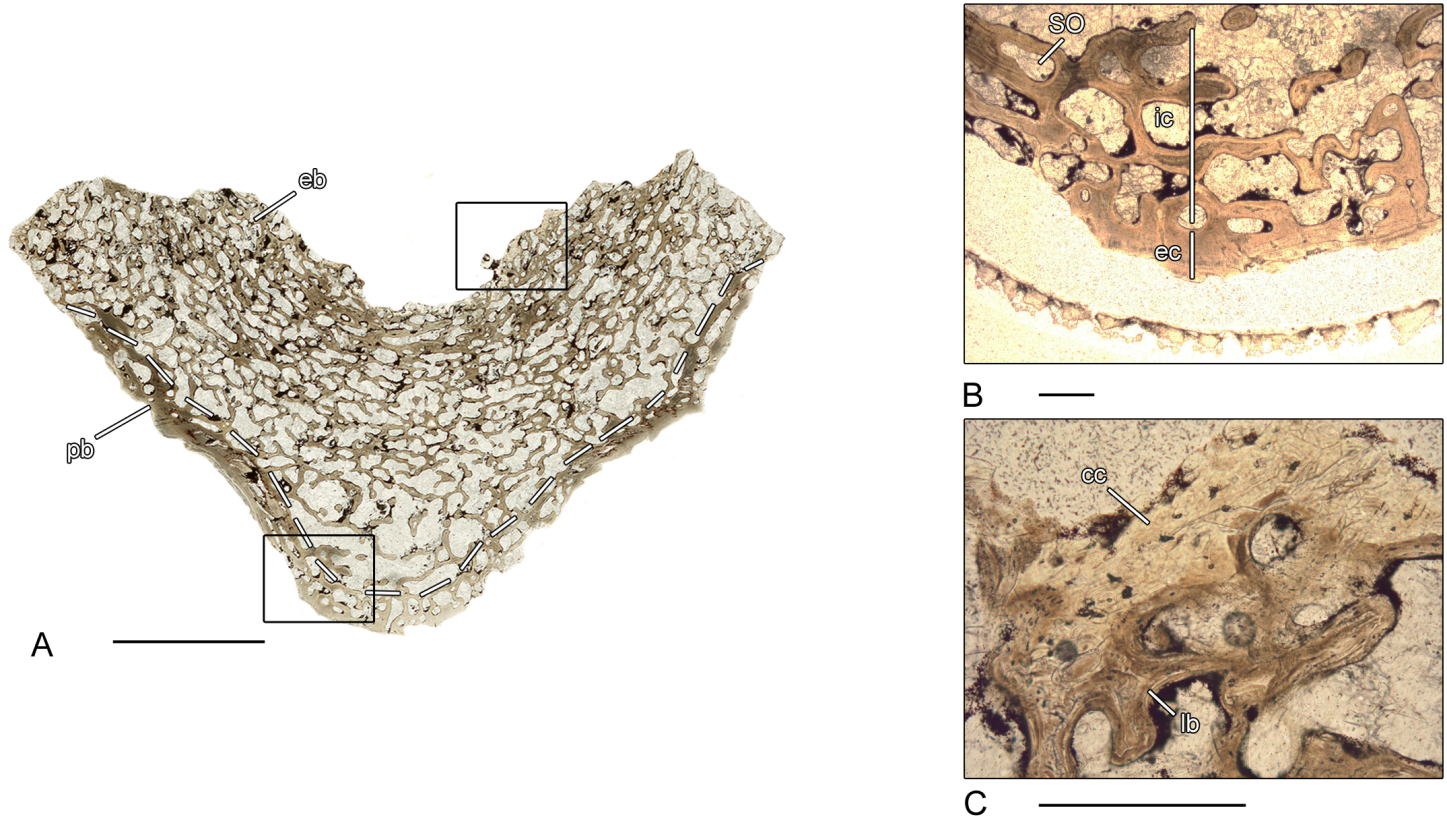
Vertebral histology of *Eryops* sp. (Temnospondyli, Eryopoidae). (A) Transverse section (1529–1) of an intercentrum of *Eryops* sp. (MB.Am.1529). (B) Close-up of (A), in normal transmitted light; two-parted periosteal cortex with a compact external layer and an internal layer of spongy, parallel-fibered bone. (C) Close-up of (A), in normal transmitted light; layer of calcified cartilage on the dorsal surface of the intercentrum. Dotted line in (A) indicates the border between periosteal and endochondral bone. In the centrum, dorsal is to the top. Scale bar in (A) equals 5mm, in (B) and (C) 500μm. Abbreviations: cc- calcified cartilage; eb—endochondral bone; ec—external cortex; ic—internal cortex; lb—lamellar bone; pb—periosteal bone; SO—secondary osteon.

In contrast to *Whatcheeria*, two distinct layers can be distinguished in the periosteal cortex of the crescentic intercentrum (Fig 4A and 4B). The external region of the cortex consists of compact, lamellar bone that is penetrated by scattered, short Sharpey’s fibers. The external layer progresses to an internal layer of spongy, parallel-fibered bone. Bone remodeling has resulted in irregularly shaped erosion rooms and a few isolated secondary osteons. The thin trabeculae in the endochondral region form a disorganized network. They consist of parallel-fibered bone with remnants of calcified cartilage in the core of the trabeculae. The trabeculae are covered with a layer of secondary bone deposit. Similar to the rhachitomous intercentrum of *Whatcheeria*, the dorsal surface is covered by a thin layer of calcified cartilage (Fig 4C).

### *Metoposaurus* sp. (MB.Am. 1437.2–2)–Temnospondyli, Stereospondyli, Metoposauridae

**Fig 5:**
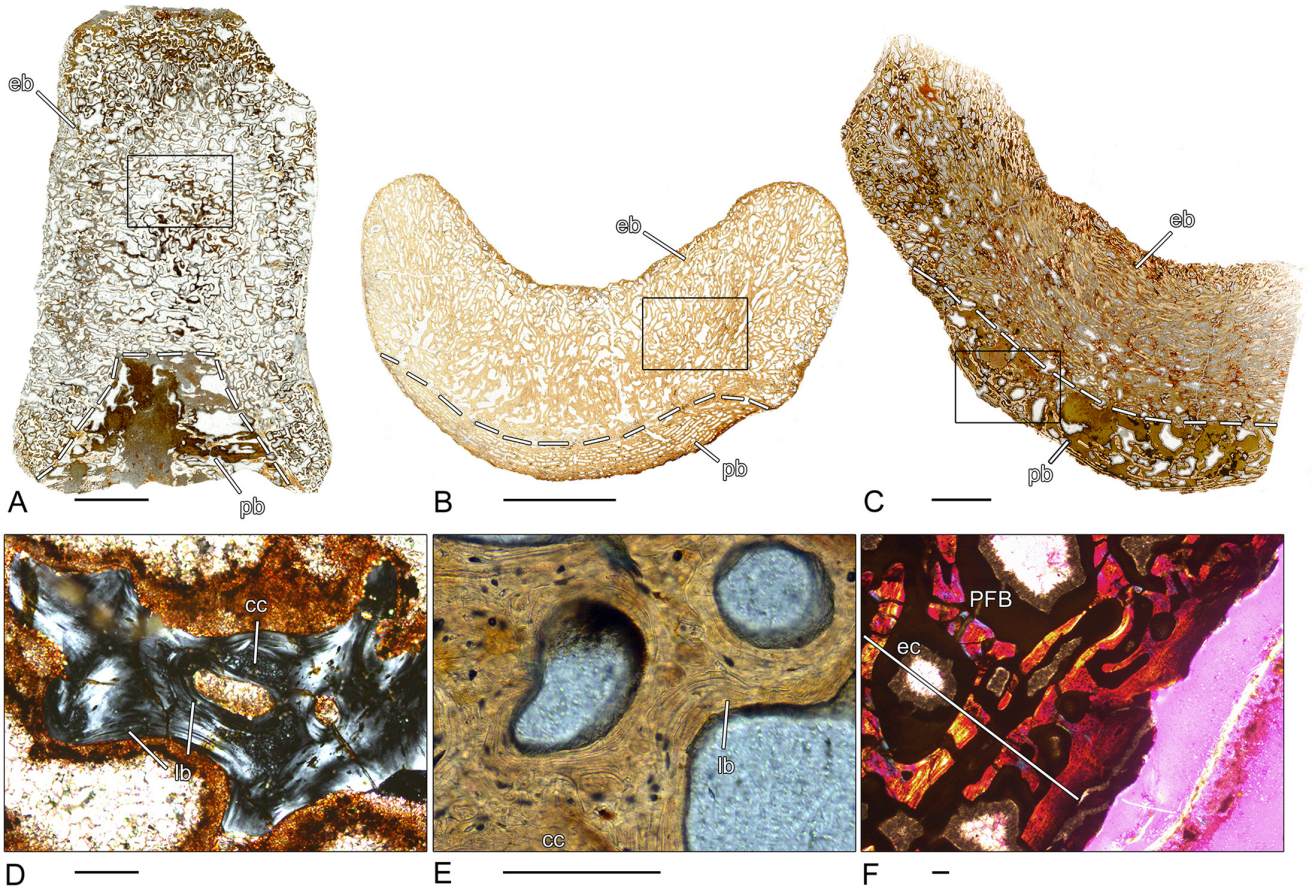
Vertebral histology of *Metoposaurus* sp., *Mastodonsaurus* sp., and *Cyclotosaurus intermedius* (Temnospondyli, Stereospondyli). (A) Longitudinal section (1437.2–2) of an intercentrum of *Metoposaurus* sp. (MB.Am.1437.2). (B) Transverse section (84213–1) of an intercentrum of *Mastodonsaurus* sp. (SMNS 84213A). (C) Transverse section (1437.11–1) of an intercentrum of *Cyclotosaurus intermedius* (MB.Am.1437.11). (D) Close-up of (A), in polarized light; remodeled trabecula that contains a core of primary calcified cartilage. (E) Close-up of (B), in normal transmitted light; remnants of calcified cartilage in the core of a trabecula. (F) Close-up of (C), in polarized light; spongy periosteal bone. Dotted line in (A)-(C) indicates the border between periosteal and endochondral bone. In all centra, dorsal is to the top. Scale bars in (A)-(C) equal 5mm, in (D)-(F) 100μm. Abbreviations: cc—calcified cartilage; eb—endochondral bone; ec—external cortex; lb—lamellar bone; pb—periosteal bone; PFB—parallel fibered bone.

In longitudinal section, the periosteal region forms a triangular shaped region and is only incompletely preserved (Fig 5A). The cancellous endochondral region is well preserved and forms the major part of the disc-shaped vertebra. The trabeculae consist of parallel-fibered bone and are densely arranged. They show varying stages of resorption and remodeling and frequently contain a core of primary calcified cartilage (Fig 5D). The amount of calcified cartilage is higher than in the vertebral centrum of *Eryops*.

### *Plagiosuchus* sp. (SMNS 52266–1; 52266–3; 52266–5)–Temnospondyli, Stereospondyli, Plagiosauridae

**Fig 6:**
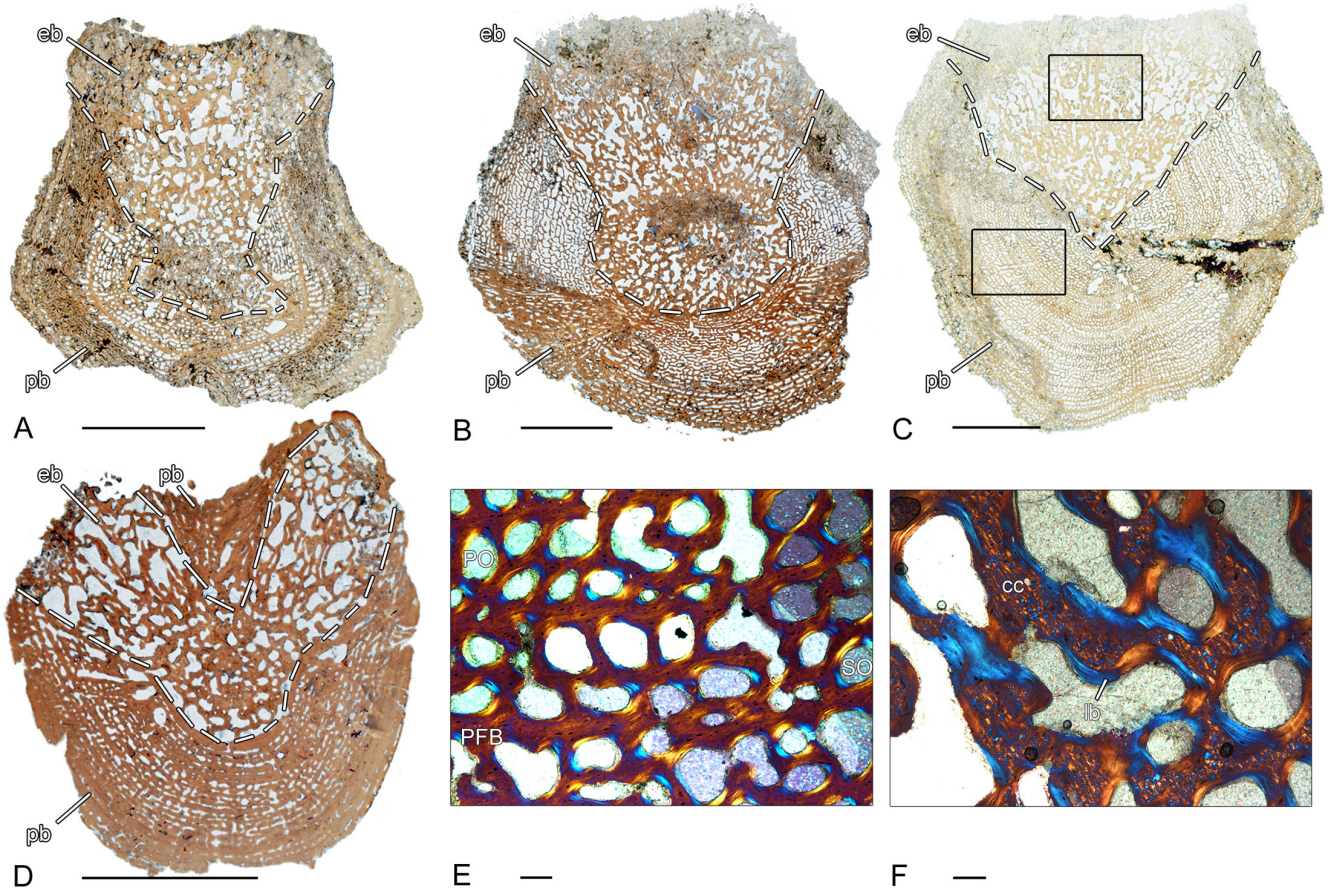
Vertebral histology of *Plagiosuchus* sp. and *Gerrothorax* sp. (Temnospondyli, Stereospondyli, Plagiosauridae). (A) Transverse section (52266–1) of a centrum of *Plagiosuchus* sp. (SMNS 52266A). (B) Transverse section (52266–3) of a centrum of *Plagiosuchus* sp. (SMNS 52266B). (C) Transverse section (52266–5) of a centrum of *Plagiosuchus* sp. (SMNS 52266C). (D) Transverse section (83451–3) of a centrum of *Gerrothorax* sp. (SMNS 83451B). (E) Close-up of (C), in polarized light; highly vascularized structure of the periosteal region. (F) Close-up of (C), in polarized light; remnants of calcified cartilage in the core of trabeculae of the endochondral region. Dotted line in (A)-(D) indicates the border between periosteal and endochondral bone. In all centra, dorsal is to the top. Scale bars in (A)-(D) equal 5mm, in (E)-(F) 100μm. Abbreviations: cc—calcified cartilage; eb—endochondral bone; lb—lamellar bone; pb—periosteal bone; PFB—parallel fibered bone; PO—primary osteon; SO—secondary osteon.

From *Plagiosuchus*, three cylindrical vertebral centra of different sizes and probably different ontogenetic stages were examined. All three vertebrae are characterized by a distinct microanatomy (Fig 6A–6C). Already in the small specimen (Fig 6A), the periosteal region is thickened and the parallel-fibered bone has a highly vascularized and spongy structure. The different types of vascular canals are mostly arranged in orderly circular layers around the center of the bone. The number of primary and secondary osteons increases from the smallest to the largest specimen (Fig 6E). Cyclically arranged bands of poorly vascularized bone display growth cycles. The endochondral region is characterized by a disorganized network of broad trabeculae formed mostly by calcified cartilage (Fig 6F). Only the surfaces have been successively remodeled and are composed of secondary lamellar bone. In all three vertebral centra, the relative size of the endochondral region is always smaller than the relative size of the periosteal region. In the smallest specimen (Fig 6A), the endochondral bone expands into the center of the vertebra, whereas in the largest specimen (Fig 6C), the endochondral bone is restricted to the dorsal part.

### *Gerrothorax* sp. (SMNS 83451–3)–Temnospondyli, Stereospondyli, Plagiosauridae

The microstructure of the cylindrical vertebral centrum is almost identical to *Plagiosuchus* (Fig 6D). The thickened periosteal bone forms the ventrolateral border of the vertebra and has a highly vascularized and spongy structure. Externally, simple vascular canals predominate, whereas internally the number of primary and secondary osteons increases. The periosteal area is penetrated by numerous Sharpey’s fibers. The trabeculae of the endochondral region form a disorganized network and, similar to *Plagiosuchus*, a high amount of calcified cartilage is preserved in the core of the trabeculae. Secondary bone is deposited on their surfaces. As seen in the undetermined adelogyrinid described above, the floor of the spinal canal is built of compact periosteal bone.

### *Mastodonsaurus* sp. (SMNS 84213–1)–Temnospondyli, Stereospondyli, Capitosauroidea

The investigated intercentrum does not have the disc-shaped morphology typical for *Mastodonsaurus* but instead is crescent-shaped (Fig 5B). Therefore, it belongs either to a not yet fully-grown trunk vertebra or to an anterior caudal intercentrum [70]. In comparison to *Eryops*, the periosteal region has an exclusively spongy structure. The mostly laminar and simple vascular canals are orderly arranged in circumferential rows. Internally, the number of primary and secondary osteons increases. The organization of the endochondral region is similar to the vertebral centrum of *Metoposaurus* and consists of parallel fibered bone with a large amount of calcified cartilage within the trabeculae (Fig 5E). The dorsal surface of the intercentrum is covered by remnants of calcified cartilage.

### *Cyclotosaurus intermedius* (MB.Am. 1437.11–1)–Temnospondyli, Stereospondyli, Capitosauroidea

Periosteal bone forms the ventro-lateral border of the crescentic intercentrum (Fig 5C and 5F). Similar to *Mastodonsaurus*, it has an exclusively spongy structure. However, the size of the intertrabecular spaces increases towards the center. The structure gets less dense and secondary bone deposits can be observed. The vascular canals are circularly orientated with only a few primary and secondary osteons. Endochondral bone forms a dense and disorganized network of thin trabeculae. Some trabeculae contain a core of calcified cartilage. The amount is lower than in the vertebral centra of *Plagiosuchus* or *Gerrothorax*. Bone remodeling is present and secondary bone is deposited on the surface of the trabeculae.

### *Archeria* sp. (MB.Am. 1533–1)–Anthracosauria, Embolomeri

**Fig 7:**
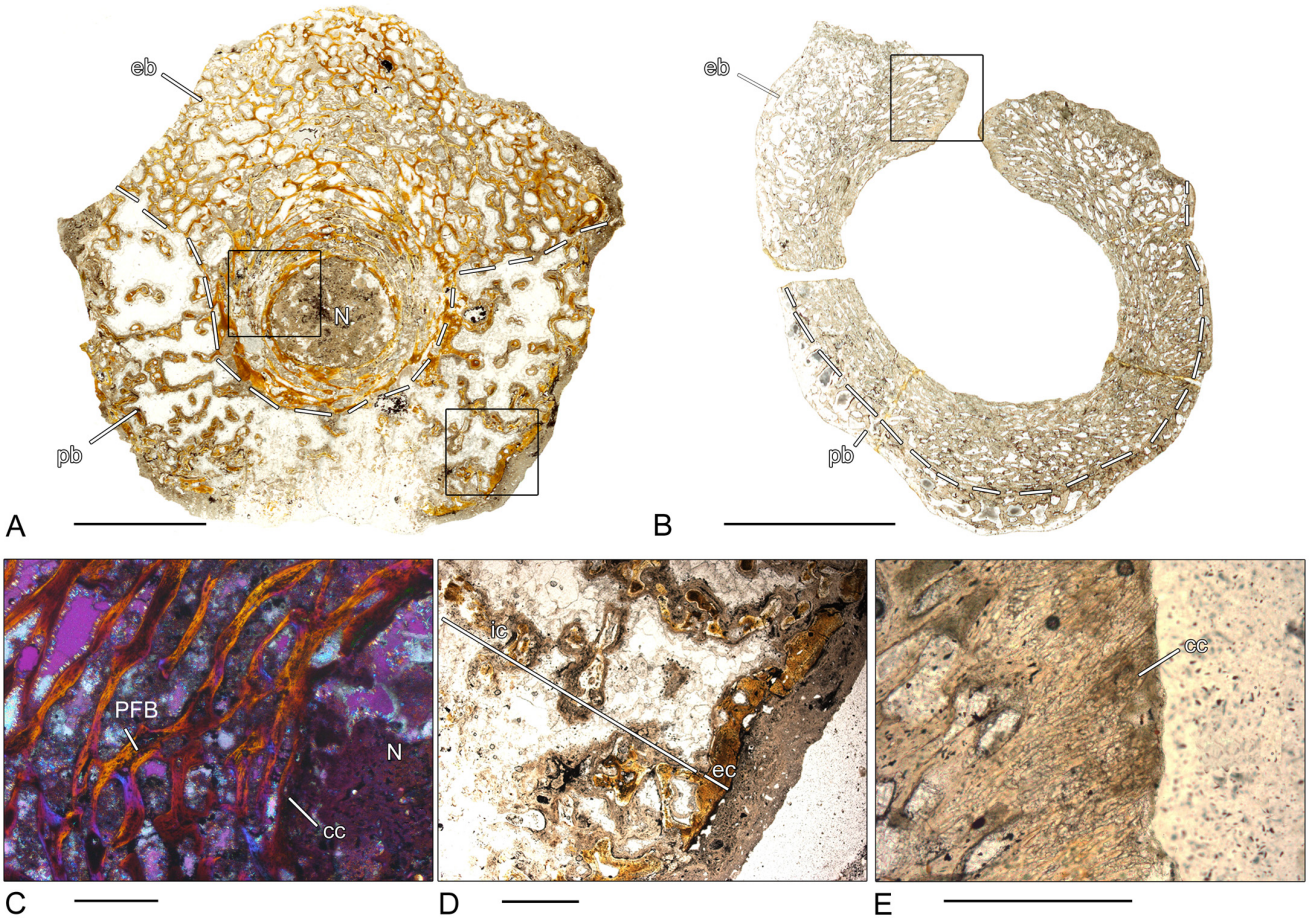
Vertebral histology of *Archeria* sp. and *Proterogyrinus* sp. (Anthracosauria, Embolomeri). (A) Transverse section (1533–1) of an indetermined centrum of *Archeria* sp. (MB.Am. 1533). (B) Transverse section (1969.4-1P) of a pleurocentrum of *Proterogyrinus* sp. (CMNH 1969–4). (C) Close-up of (A), in polarized light; notochordal canal surrounded by bone arranged in circular rows. (D) Close-up of (A), in normal transmitted light; two-parted periosteal cortex with a compact external layer and a loose internal layer. (E) close-up of (B), in normal transmitted light; high amount of calcified cartilage in the dorsal peaks of the pleurocentrum. The dotted line in (A) and (B) indicates the border between periosteal and endochondral bone. In all centra, dorsal is to the top. Scale bars for (A) and (B) equal 5mm, (C)-(E) equal 500μm. Abbreviations: cc—calcified cartilage; eb—endochondral bone; ec—external cortex; ic—internal cortex; N—notochordal canal; pb—periosteal bone; PFB—parallel fibered bone.

Because of the lack of specific morphological features, it could not be determined if the present disk-shaped element is an intercentrum or pleurocentrum (Fig 7A). Similar to the Plagiosauridae, the periosteal region is thickened, but the composition of this region differs distinctly from the former group. Here, the periosteal region can be subdivided into a compact external layer of lamellar bone and an internal layer of loose, spongy parallel-fibered bone (Fig 7D). Lines of resorptions and secondary bone deposits can be observed in the internal layer. Laterally, a number of short, isolated Sharpey’s fibers are visible. The endochondral dorsal section of the centrum consists of dense cancellous bone. The trabeculae are strongly remodeled and secondary lamellar bone can be identified on their outer surfaces. Remnants of calcified cartilage are very rare. The centrally located notochordal canal is surrounded by secondary remodeled bone that is arranged in circular rows (Fig 7C). The outline of the internal surface of the canal has irregularly dispersed remnants of calcified cartilage.

### *Proterogyrinus* sp. (CMNH 1969.4-1P)–Anthracosauria, Embolomeri

The periosteal cortex of the horseshoe-shaped pleurocentrum of *Proterogyrinus* is two-layered, similar to the intercentrum of *Eryops* and the centrum of *Archeria* described above (Fig 7B). The external layer is thin and composed of compact, lamellar bone. The internal layer has a spongy structure with large erosion rooms. The mostly primary bone consists of parallel fibered bone matrix. Laterally, a few Sharpey’s fibers are preserved. The thin trabeculae of the endochondral region form a disorganized network with a high amount of calcified cartilage in the dorsal region (Fig 7E). However, the amount of calcified cartilage is not as high as in stereospondyls. The dorsal border is covered by a thin layer of calcified cartilage. Little can be said about the degree of bone remodeling due to poor preservation of the bone matrix.

### *Discosauriscus* sp. (DE.KO. 187–1) and *D*. *austriacus* (Z.K 52–1)–Seymouriamorpha

**Fig 8:**
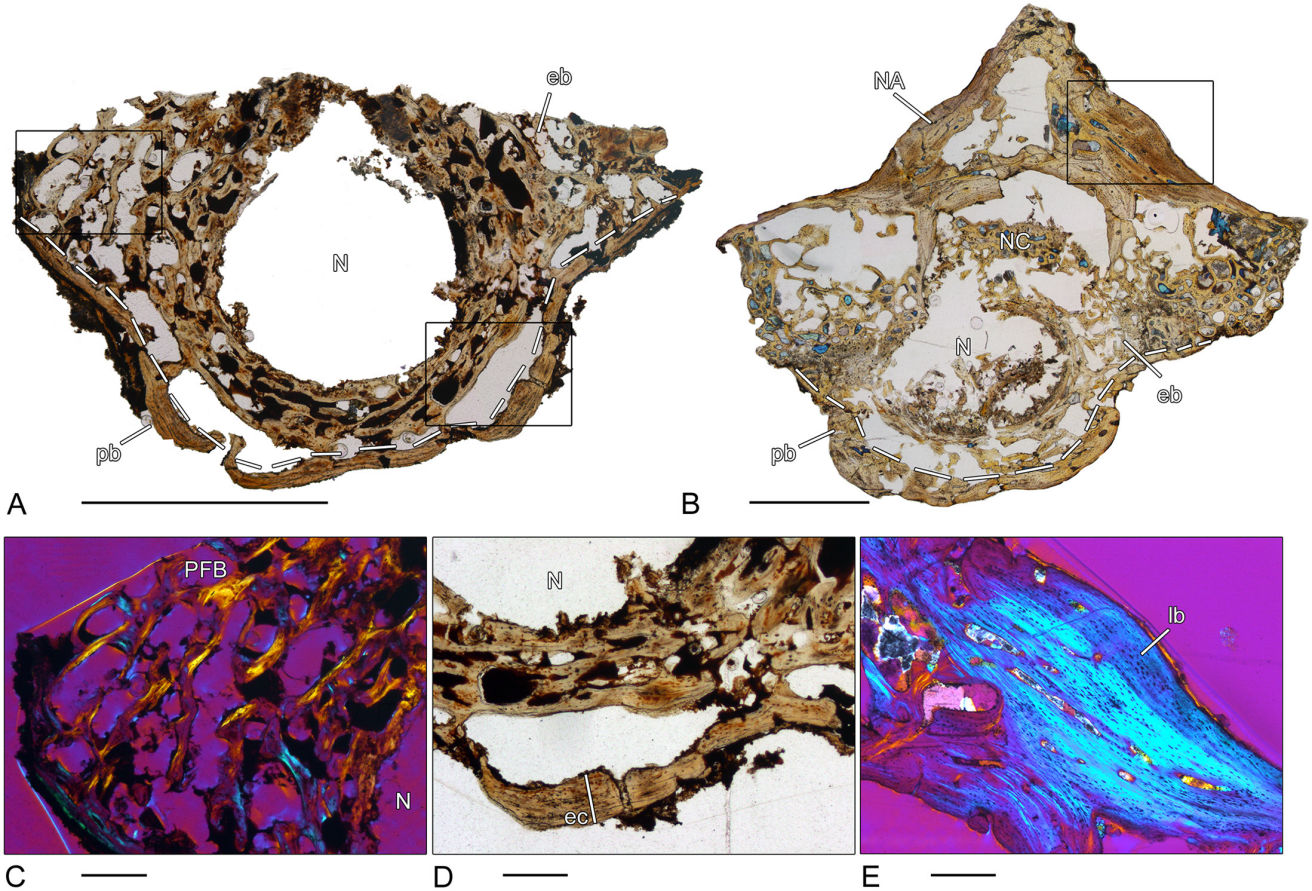
Vertebral histology of *Discosauriscus* sp. and *D*. *austriacus* (Seymouriamorpha). (A) Transverse section (KO187-1) of a pleurocentrum of *Discosauriscus* sp. (DE.KO.187). (B) Transverse section (K52-1) of a pleurocentrum of *Discosauriscus austriacus* (Z15568 [K52]). (C) Close-up of (A), in polarized light; strongly remodeled endochondral region. (D) Close-up of (A), in normal transmitted light; thin compact and nearly avascular periosteal region. (E) Close-up of (B), in polarized light; compact, lamellar bone in the neural arch. Dotted line in (A) and (B) indicates the border between periosteal and endochondral bone. In all centra, dorsal is to the top. Scale bars in (A) and (B) equal 1mm, in (C)-(E) 200μm. Abbreviations: eb—endochondral bone; ec—external cortex; lb—lamellar bone; N—notochordal canal; NA—neural arch; NC—neural canal; pb—periosteal bone; PFB—parallel fibered bone.

Two ring-shaped pleurocentra of *Discosauriscus* were studied histologically. In K52, the pleurocentrum is fused to the neural arch (Fig 8B), whereas the other pleurocentrum, DE.KO.187, is detached from it (Fig 8A). In both, a thin band of periosteal lamellar bone forms a compact ventrolateral border (Fig 8D) in which scattered, short Sharpey’s fibers can be recognized. The endochondral region forms the dorsal part of the bone. Strikingly, the trabeculae are strongly remodeled despite the relatively early ontogenetic stage of the specimens, as determined from shape and size of the vertebrae. Furthermore, the amount of calcified cartilage is very low in this region. The large notochordal canal occupies the major part of the centrum. It is surrounded by circularly arranged trabeculae of parallel-fibered bone with secondary bone deposits on their surface. Remnants of calcified cartilage are visible on the internal surface of the notochordal canal. The neural arch, extending dorsally around the neural canal, consists of lamellar bone with a very compact and nearly avascular structure (Fig 8E).

### *Seymouria* sp. (OMNH 73499)–Seymouriamorpha

**Fig 9:**
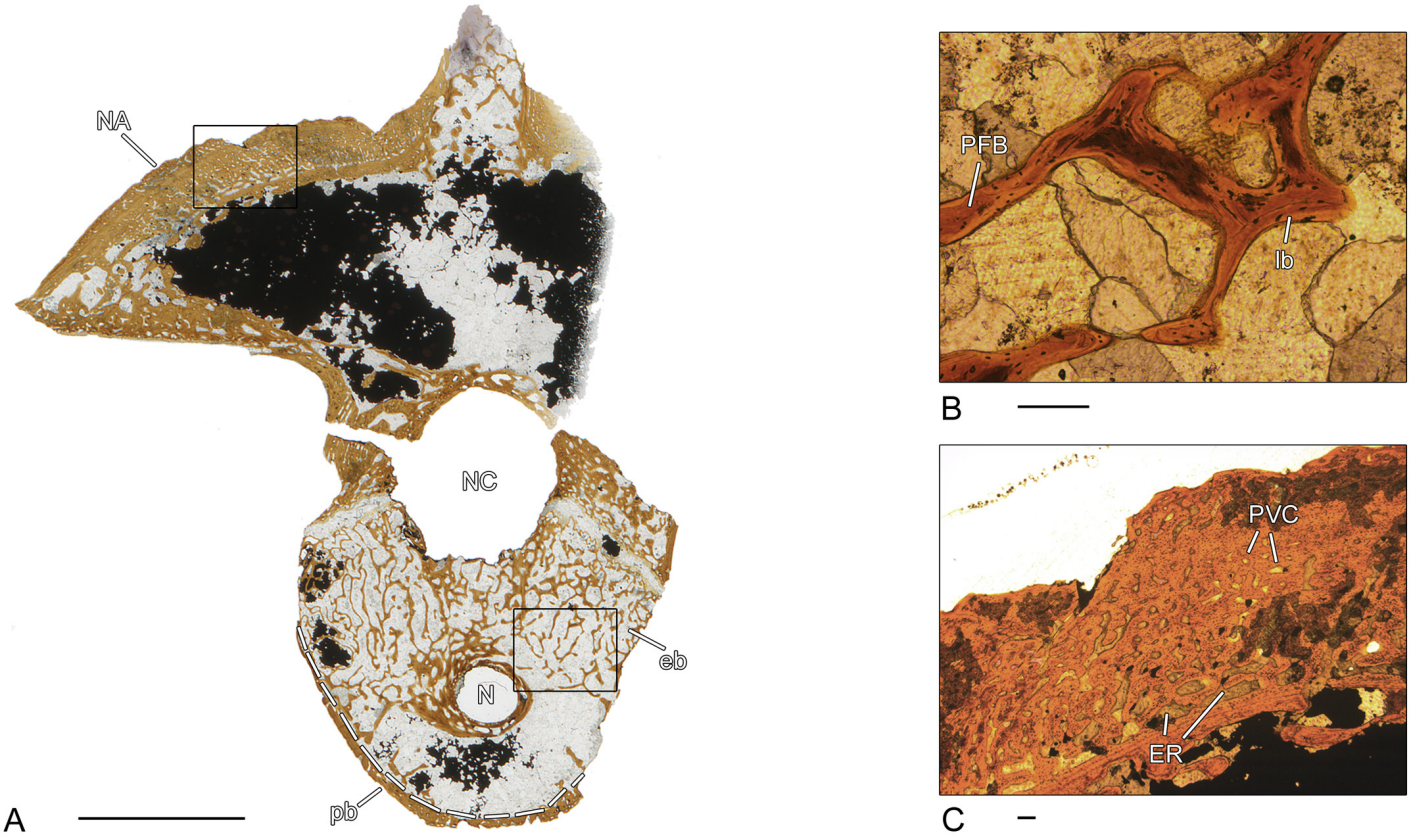
Vertebral histology of *Seymouria* sp. (Seymouriamorpha). (A) Transverse section (73499) of a pleurocentrum of *Seymouria* sp. (OMNH 73499). (B) Close-up of (A), in normal transmitted light; remodeled trabecula with secondary lamellar bone on its surface. (C) Close-up of (A), in normal transmitted light; neural arch with a highly spongy structure. Dotted line in (A) indicates the border between periosteal and endochondral bone. In the centrum, dorsal is to the top. Scale bar in (A) equals 5mm, in (B) and (C) 100μm. Abbreviations: eb—endochondral bone; ER—erosion room; lb—lamellar bone; N—notochordal canal; NA—neural arch; NC—neural canal; pb—periosteal bone; PFB—parallel fibered bone; PVC—primary vascular canal.

The cylindrical pleurocentrum of *Seymouria* is fused to the neural arch (Fig 9A). As in *Discosauriscus*, the ventrolateral border is composed of lamellar periosteal bone that forms a thin band and has a compact structure. Isolated primary and secondary osteons are present. A disorganized network of thin trabeculae forms the endochondral region. The trabeculae are remodeled and secondary lamellar bone is deposited on their surface but calcified cartilage is absent (Fig 9B). The narrow notochordal canal is surrounded by spongy bone. Similar to *Discosauriscus*, remnants of calcified cartilage are found on the internal surface of the notochordal canal. The microstructure shows a cartilaginous separation between pleurocentrum and neural arch. In contrast to *Discosauriscus*, the neural arch is lightly built with a highly spongy structure (Fig 9C).

### *Chroniosaurus dongusensis* (PIN 3585.220; 3585.214; 3585.218)–Chroniosuchia, Chroniosuchidae

**Fig 10:**
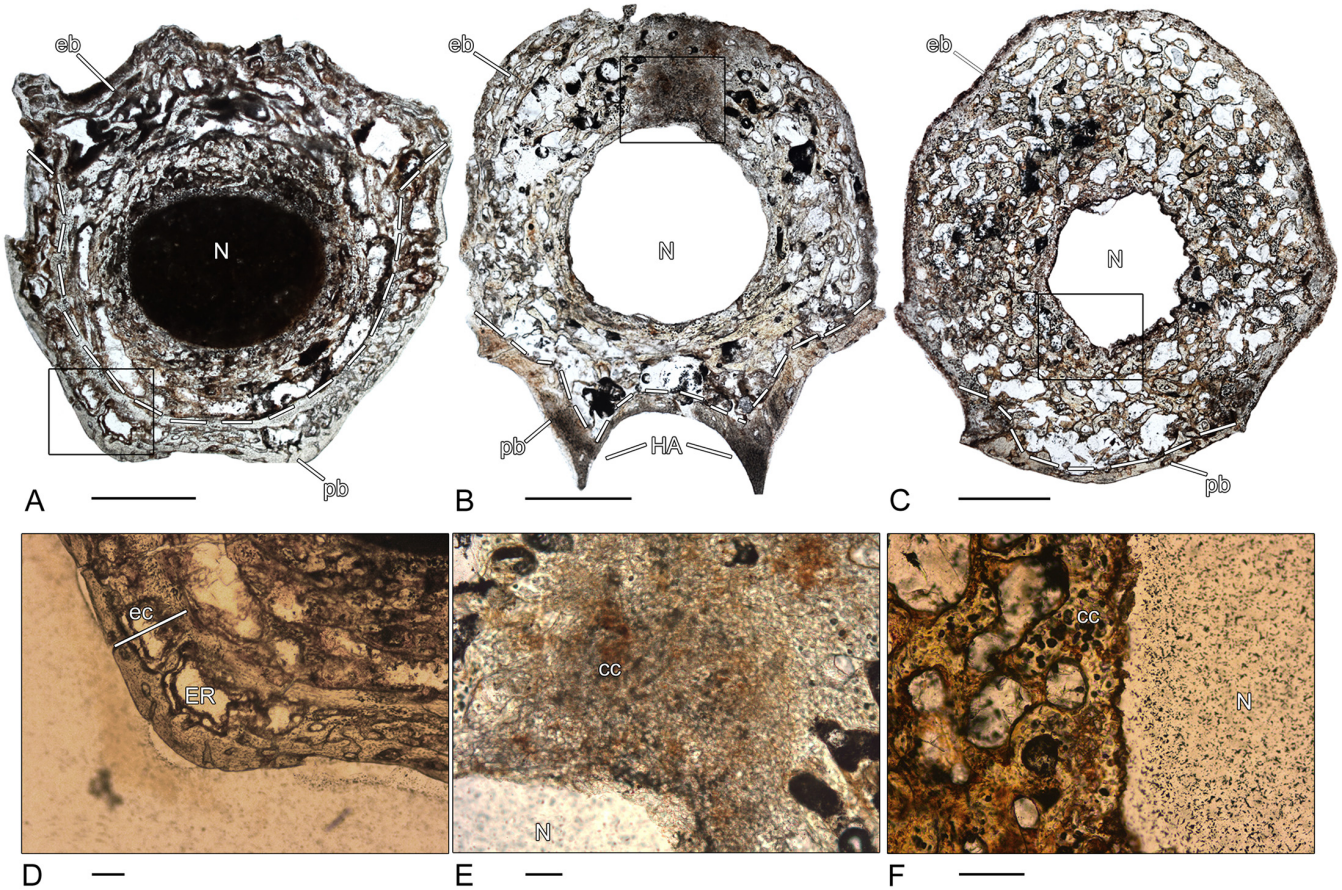
Vertebral histology of *Chroniosaurus dongusensis* (Chroniosuchia, Chroniosuchidae). (A) Transverse section (3585–220) of a pleurocentrum of *Chroniosaurus dongusensis* (PIN 3585–220). (B) Transverse section (3585–214) of an intercentrum of *Chroniosaurus dongusensis* (PIN 3585–214). (C) Transverse section (3585–218) of an intercentrum of *Chroniosaurus dongusensis* (PIN 3585–218). (D) Close-up of (A), in normal transmitted light; spongy periosteal region with large erosion rooms. (E) Close-up of B, in normal transmitted light; cartilaginous structure of the dorsal area of the intercentrum. (F) Close-up of (C), in normal transmitted light; remnants of calcified cartilage on the internal surface of the notochordal canal. Dotted line in (A)-(C) indicates the border between periosteal and endochondral bone. In all centra, dorsal is to the top. Scale bars in (A)-(C) equal 1mm, in (D)-(F) 100μm. Abbreviations: cc—calcified cartilage; eb—endochrondral bone; ec—external cortex; ER—erosion room; HA—haemal arch; N—notochordal canal; pb—periosteal bone.

Due to the small size and the ring-shaped intercentra, it is likely that the examined material belongs to juvenile specimens. The cylindrical pleurocentrum 3585.220 is characterized by a band of lamellar periosteal bone along its ventral border (Fig 10A and 10D). Scattered, simple vascular canals and erosion rooms are distributed in this area. Sharpey’s fibers are absent. The major part of the pleurocentrum is composed of endochondral bone consisting of secondary remodeled trabeculae forming a dense and disorganized network. Calcified cartilage is very rarely preserved within the core of the trabeculae. Similar to other investigated centra, the dorsal side is covered by a thin layer of calcified cartilage. The lateral sides display the articulation surfaces for the ribs and have a cartilaginous structure. The centrally located and rounded notochordal canal is surrounded by spongy bone. A relatively high amount of calcified cartilage is visible on the internal surface of the notochordal canal.

In the ring-shaped intercentrum 3585.218, the ventrally located band of compact, nearly avascular periosteal bone is very thin (Fig 10C). Almost the entire centrum is composed of a dense, disorganized network of endochondral bone. Secondary bone is deposited on the surface of the trabeculae and remnants of calcified cartilage are preserved in the core of some of them. The dorso-lateral surface is covered by a thin layer of calcified cartilage. The rounded notochord canal is centrally located and surrounded by a band of calcified cartilage (Fig 10F). In contrast to the pleurocentrum, the notochordal area is not fully distinct from the rest of the bone. The microanatomy of the caudal, ring-shaped intercentrum 3585.214 differs slightly (Fig 10B). The periosteal bone is limited to the ventral side and is distinctly thicker. The compact, lamellar bone is nearly avascular whereas the endochondral bone is composed of dense cancellous bone. Compared to the former *Chroniosaurus* intercentrum, less calcified cartilage is preserved within the trabeculae, which show varying stages of resorption and remodeling. As in 3585.218, a thin layer of calcified cartilage covers the dorso-lateral area. Interestingly, the dorsal part of the ring-shaped intercentrum has a completely cartilaginous structure (Fig 10E).

### *Bystrowiella schumanni* (SMNS 81872–1; 81872–6)–Chroniosuchia, Bystrowianidae

**Fig 11:**
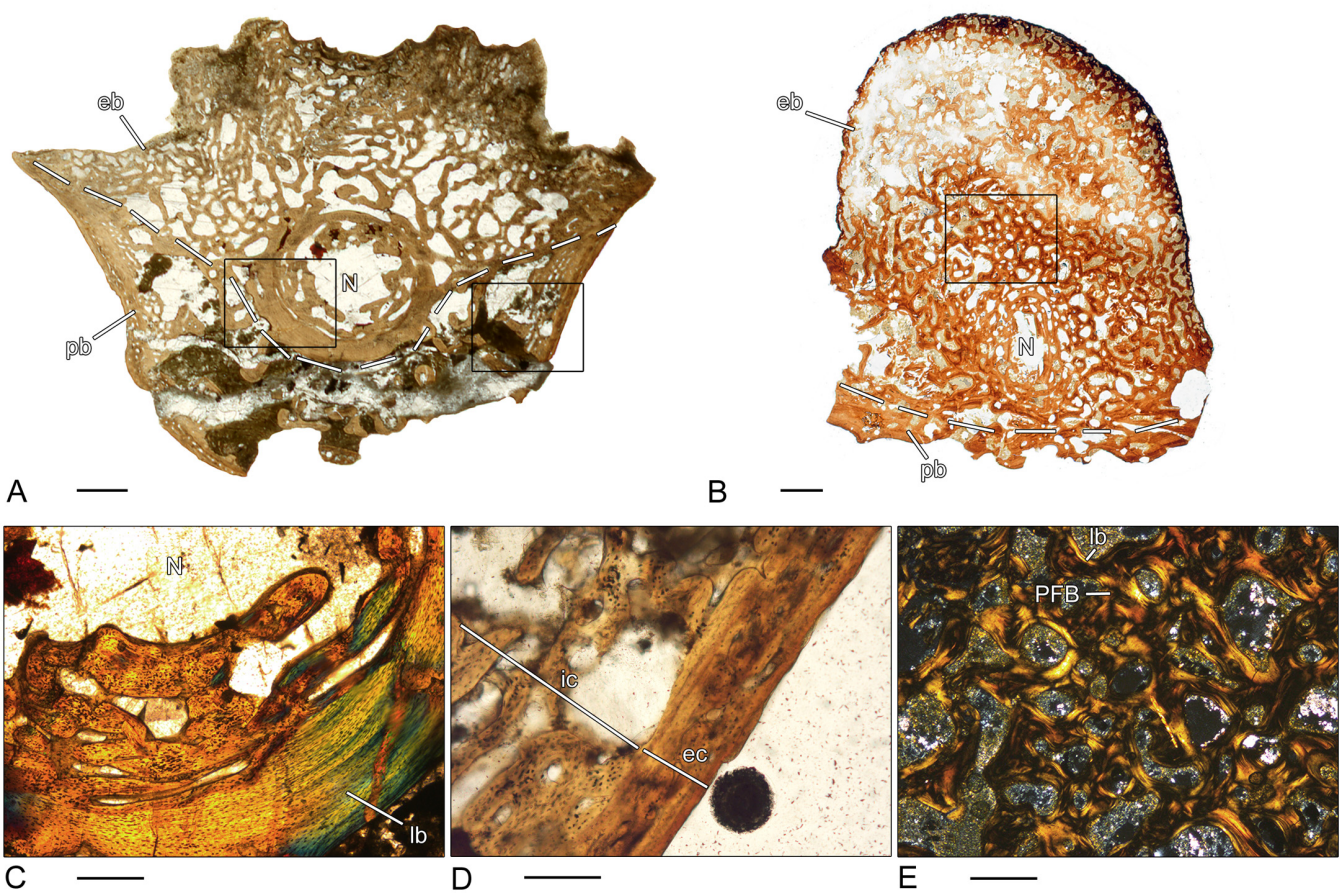
Vertebral histology of *Bystrowiella schumanni* (Chroniosuchia, Bystrowianidae). (A) Transverse section (81872–1) of a pleurocentrum of *Bystrowiella schumanni* (SMNS 81872A). (B) Transverse section (81872–6) of an intercentrum of *Bystrowiella schumanni* (SMNS 81872E). (C) Close-up of (A), in normal transmitted light; notochordal canal surrounded by a two-layered bone structure. (D) Close-up of (A), in normal transmitted light; two-layered periosteal cortex with a compact external layer and a loose internal layer. (E) Close-up of (B), in polarized light; strongly remodeled endochondral region. Dotted line in (A) and (B) indicates the border between periosteal and endochondral bone. In all centra, dorsal is to the top. Scale bars in (A) and (B) equal 1mm, in (C)-(E) 200μm. Abbreviations: eb—endochondral bone; ec—external cortex; ic—internal cortex; lb—lamellar bone; N—notochordal canal; pb—periosteal bone; PFB—parallel fibered bone.

Both an intercentrum and pleurocentrum of *Bystrowiella* were examined. The microanatomy of the cylindrical pleurocentrum resembles that of the above-described centrum of *Archeria* (Fig 11A). The thickened periosteal region consists of a compact, external layer of lamellar bone and a loose, spongy internal region (Fig 11D). The degree of bone remodeling is low in the external layer, which is mainly composed of primary bone. In contrast, the internal layer is highly remodeled. The endochondral bone is parallel-fibered and forms a cancellous structure of thin trabeculae in the dorsal part of the pleurocentrum. Deposits of secondary bone can be observed, and only a few remnants of calcified cartilage are preserved within the cores of the trabeculae. In contrast to *Chroniosaurus*, the centrally located notochordal canal is surrounded by bone in which two layers can be distinguished: the compact external layer forms a complete ring of lamellar, primary bone that is avascular and displays a large number of osteocyte lacunae, while the internal layer has a more trabecular structure and bone remodeling can be observed (Fig 11C). Remnants of calcified cartilage cannot be found on the internal surface of the notochordal canal.

The microanatomy of the ball-shaped intercentrum differs slightly from that of the pleurocentrum of *Bystrowiella* (Fig 11B). The periosteal region is thin, restricted to the ventral border and penetrated by a large number of Sharpey’s fibers. The endochondral region is extended and occupies almost the entire surface area of the intercentrum. The parallel-fibered trabeculae form a dense and disorganized network. The trabeculae are strongly remodeled and only few remnants of calcified cartilage are visible in their core (Fig 11E). A thin layer of calcified cartilage covers the dorso-lateral surface of the centrum. The notochordal canal penetrates the intercentrum, but is distinctly smaller than in the pleurocentrum. Moreover, the canal is surrounded only by cyclically arranged trabecular bone. Similar to the *Chroniosaurus* intercentrum, the notochordal area is not as delimited from the endochondral region as in the pleurocentrum. No calcified cartilage is observed on the internal surface of the canal.

### Nectridea indet. (FMNH UR 2511)–Lepospondyli, Nectridea

**Fig 12:**
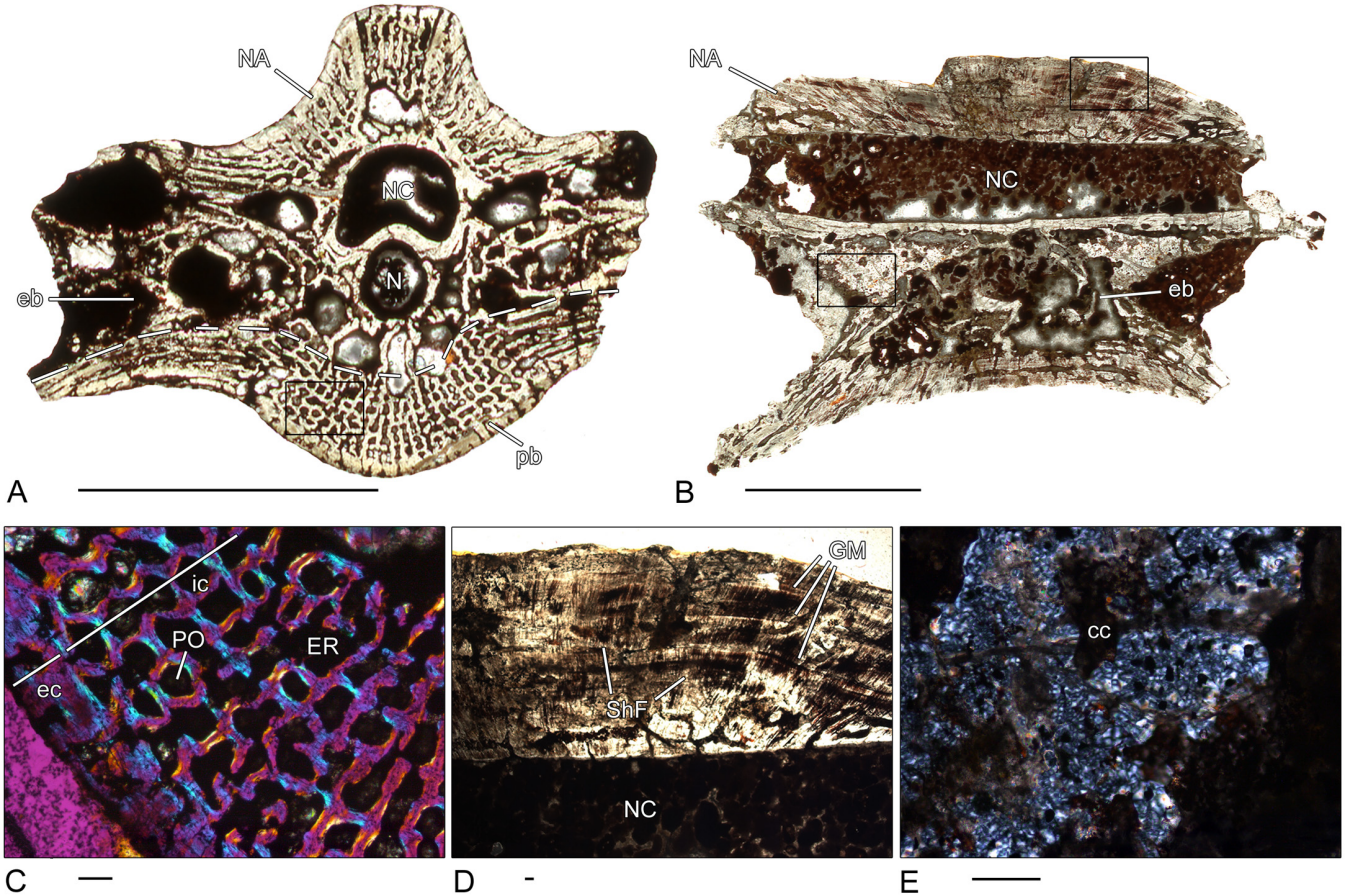
Vertebral histology of Nectridea indet. and *Diplocaulus magnicornis* (Lepospondyli, Nectridea). (A) Transverse section (2511) of a pleurocentrum of Nectridea indet. (FMNH UR 2511). (B) Longitudinal section (2510) of a pleurocentrum of *Diplocaulus magnicornis* (FMNH UR 2510). (C) Close-up of (A), in polarized light; two-layered periosteal cortex with a compact external layer and a spongy internal layer. (D) Close-up of (B), in normal transmitted light; neural arch penetrated by numerous Sharpey’s fibers. (E) Close-up of (B), in polarized light; remnants of calcified cartilage on the concave articular surfaces of the centrum. Dotted line in (A) indicates the border between periosteal and endochondral bone. In all centra, dorsal is to the top. Scale bars in (A) and (B) equal 5mm, in (C)-(E) 100μm. Abbreviations: cc—calcified cartilage; eb—endochondral bone; ec—external cortex; ER—erosion room; GM—growth mark; ic—internal cortex; N—notochordal canal; NA—neural arch; NC—neural canal; pb—periosteal bone; PO—primary osteon; ShF—Sharpey’s fibers.

The ventro-lateral area of the spool-shaped centrum is composed of thickened, two-layered, periosteal bone (Fig 12A and 12C). The thin external layer has a compact structure and progresses into a thicker, internal layer of spongy bone. The internal layer is highly vascularized with primary and secondary osteons and large erosion rooms. The region of periosteal bone consists of parallel-fibered bone matrix. Secondary bone depositions can be observed. The endochondral region has a highly cancellous structure without calcified cartilage. The notochordal canal is located centrally and surrounded by a thin band of compact, lamellar bone. The internal surface of the canal is irregular. Similar to *Seymouria*, the neural arch is thickened and has a spongy structure. The bone is speckled by erosion rooms and vascular canals.

### *Diplocaulus magnicornis* (FMNH UR 2510)–Lepospondyli, Nectridea

The spool-shaped vertebral centrum of *Diplocaulus* has been sectioned longitudinally (Fig 12B). The ventral border is incompletely preserved, but the periosteal bone in this region appears to have a spongy structure. A loose network of endochondral trabeculae of parallel-fibered bone is present in the center of the centrum. Large areas of calcified cartilage are preserved in the concave (amphicoelous) articular surfaces of the centrum (Fig 12E). The neural arch is composed of compact, lamellar, and nearly avascular bone and is penetrated by numerous Sharpey’s fibers (Fig 12D). Additionally, at least three growth marks can be found in the dorsal part of the neural arch.

### *Microbrachis* sp. (MB.Am. 854–1)–Lepospondyli, “Microsauria”

**Fig 13:**
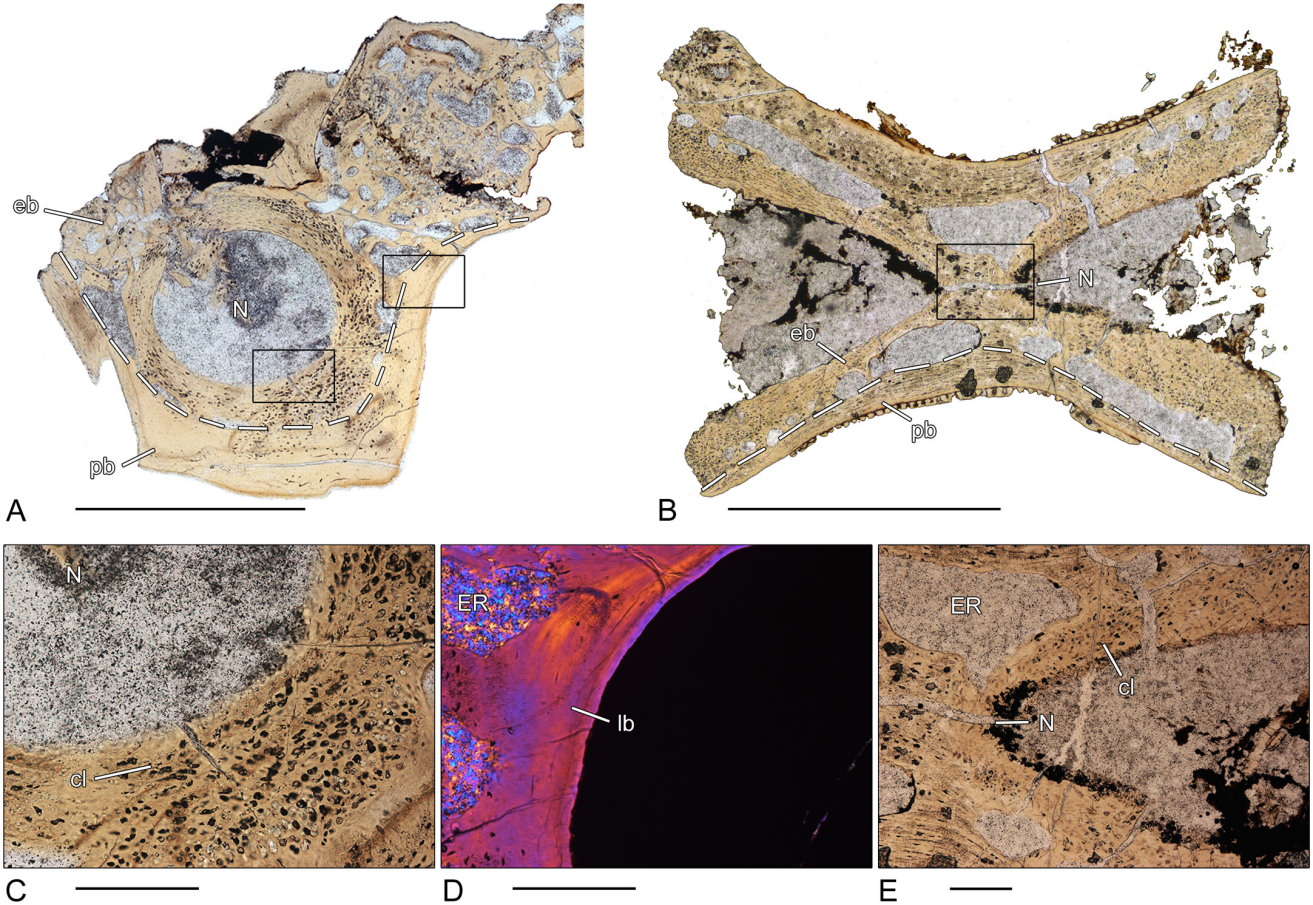
Vertebral histology of *Microbrachis* sp. (Lepospondyli, “Microsauria”). (A) Transverse section (854–1) of a pleurocentrum of *Microbrachis* sp. (MB.Am.854). (B) Longitudinal section (865) of a pleurocentrum of *Microbrachis* sp. (MB.Am.865). (C) Close-up of (A), in normal transmitted light; notochordal canal surrounded by large cell lacunae. (D) Close-up of (A), in polarized light; compact, avascular lamellar periosteal bone. (E) Close-up of (B), in normal transmitted light; concave articular surfaces composed of large cell lacunae. Dotted line in (A) and (B) indicates the border between periosteal and endochondral bone. In all centra, dorsal is to the top. Scale bars in (A) and (B) equal 1mm, in (C)-(E) 200μm. Abbreviations: cl- cell lacuna; eb—endochondral bone; ER—erosion room; lb—lamellar bone; N—notochordal canal; pb—periosteal bone.

The spool-shaped vertebral centrum of *Microbrachis* has been sectioned transversally (Fig 13A). Similar to *Doleserpeton*, the periosteal bone has a compact, avascular structure (Fig 13D). It forms the ventrolateral border of the centrum and consists of lamellar bone. The rather compact, small endochondral region is located latero-dorsally and consists of broad trabeculae. The centrally located notochordal canal is large and occupies a substantial part of the centrum. Similar to *Doleserpeton*, the canal is surrounded by large cell lacunae (Fig 13C). The suture between centrum and the remnant of the neural arch has a cartilaginous structure.

### *Microbrachis* sp. (MB.Am. 865)–Lepospondyli, “Microsauria”

This centrum has been longitudinally sectioned and its structure is similar to the other *Microbrachis* sp. centrum MB.Am.854 and to the pleurocentrum of *Doleserpeton* (OMNH 73530F) (Fig 13B). The periosteal bone consists of compact lamellar bone externally and is nearly avascular. Similar to MB.Am.854, the region has a very compact structure. As seen in *Doleserpeton*, the concave (amphicoelous) articular surfaces of the centrum are characterized by a broad band of large cell lacunae (Fig 13E). The neural arch, normally suturally attached to the centrum, is incompletely preserved.

## Discussion

**Fig 14:**
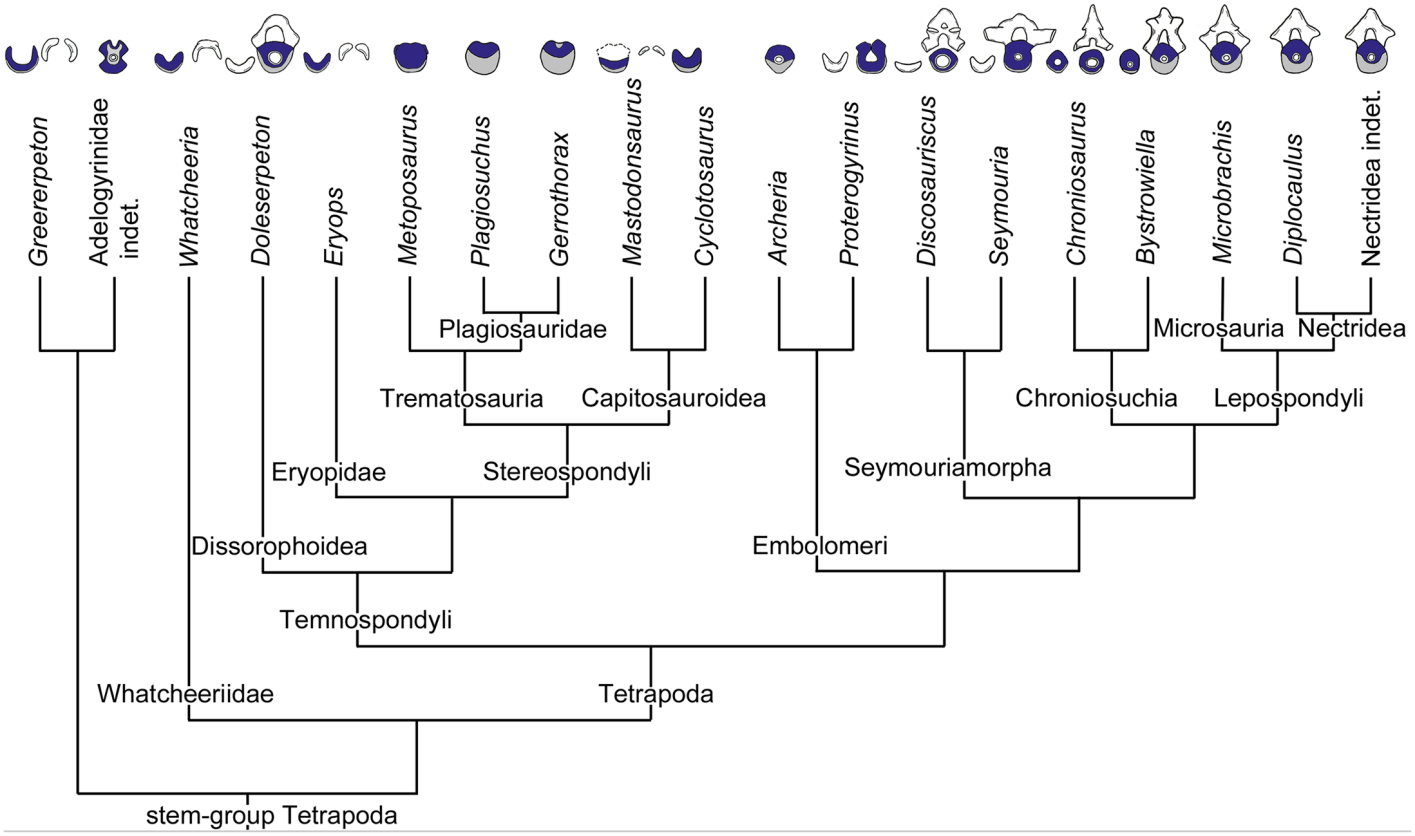
Distribution of periosteal and endochondral bone in the vertebral centra investigated in this study. After [4, 10, 65, 66]. The sizes of the vertebral elements are not to scale and have been redrawn from [16, 21, 24, 34, 41, 67–73]. Grey: periosteal bone; blue: endochondral bone.

The results of our histological studies of the vertebral centra of different Paleozoic and Mesozoic basal tetrapods indicate that regardless of whether the centra are monospondylous (i.e. lepospondyls) or multipartite (i.e. most stem-tetrapods, temnospondyls, anthracosaurs, and seymouriamorphs), they ossify both endochondrally and perichondrally/periosteally (Fig 14). Two ossification patterns of the vertebral centra can be distinguished and are described below.

### Ossification processes

The vertebral centrum (or centra in multipartite vertebrae) is formed initially as cartilaginous element(s). As ontogeny progresses, the cartilaginous trabeculae are replaced by parallel-fibered bone, and lamellar endosteal bone is deposited on the surface of the trabeculae. The resulting endochondral bone forms a disorganized, cancellous network that persists in centra of adult individuals, where it forms the dorso-central portion. Periosteal bone is centrifugally deposited on the ventro-lateral side on the outer surface of the centrum. Konietzko-Meier et al. [63] demonstrated that periosteal ossification occurs after endochondral ossification in vertebral centra development of temnospondyls. All centra investigated in the present study possess a portion of periosteal bone, independent of their ontogenetic stage. In the presumed juvenile specimens of *Discosauriscu*s and *Chroniosaurus*, for example, a small strip of compact periosteal bone already exists in the ventro-lateral part of the pleuro- and intercentrum (Figs 8A–8B and 10A–10C).

The endochondral ossification contributes to both the longitudinal and centrifugal growth of the centra, the periosteal ossification, however, only to the latter. The extent of the two ossification types and their underlying developmental processes differ conspicuously among the vertebral centra of the taxa described here. For example, the centrifugal growth of the ring-shaped pleurocentrum of *Proterogyrinus* is mainly the result of endochondral ossification, with the ossification of the trabeculae being more advanced in the ventral part of the centrum, whereas in the dorsal part, a larger amount of calcified cartilage is present (Fig 7B and 7E). Therefore, the dorsal portion of the centra can be regarded as ontogenetically younger. The periosteal region of *Proterogyrinus* pleurocentra is thin and limited to the ventro-lateral side. These histological data agree with morphological observations by Holmes [14]: in small individuals of *Proterogyrinus*, the pleurocentrum is dorsally open and U-shaped, but it subsequently continues to ossify until the dorsal gap is closed. Further examples of vertebral elements with a predominantly endochondral ossification are the intercentra of *Whatcheeria* (Fig 2C), *Mastodonsaurus* (Fig 5B), and *Bystrowiella* (Fig 11B). The extent of endochondral ossification is therefore independent of the vertebral segment (intercentrum vs. pleurocentrum) or the phylogenetic position (stem-tetrapod, temnospondyls, chroniosuchian).

The centrifugal growth of the vertebral centra of the Plagiosauridae, however, results from a predominance of periosteal ossification. Analysis of different growth stages of plagiosaurid material showed that the relative thickness of the periosteal region increases, whereas the endochondral region remains approximately equally thick. In the largest specimen of *Plagiosuchus*, almost the entire centrum is formed by periosteal bone (Fig 6C). A similar predominance of periosteal ossification can be observed in the vertebral centra of *Archeria* (Fig 7A), Nectridea indet. (Fig 12A), *Microbrachis* (Fig 13A), and *Doleserpeton* (Fig 3A).

Strikingly, most of these taxa ossify their vertebral centra at an early ontogenetic stage and possess disc-like or spool-shaped centra. The stem-stereospondyl *Archegosaurus* exemplified the slow skeletal development of some aquatic stereospondylomorphs [75]: skeletal elements such as the vertebral centra, neurocranium, or scapulacoracoid ossify relatively late. In comparison, the Plagiosauridae developed their skeleton at a faster rate: in a medium-sized *Gerrothorax* specimen (skull length 75mm), the vertebral centra are already ossified and the pectoral girdle is remarkably similar to fully grown forms [36, 76]. Schoch [77] argued that this fast development protected these immobile animals against other predators. Hence, vertebral development in the Plagiosauridae was achieved by a predominantly periosteal ossification with a fast growth rate. Histologically, this fast growth rate is indicated by spongy, highly vascularized, fibro-lamellar bone [51, 78].

As mentioned in the introduction, the monospondylous vertebral centra of lepospondyls ossified at a very early ontogenetic stage. In a very small specimen of the “microsaur” *Hyloplesion* (16mm total body length), the vertebral centra already form thin-walled cylinders [44]. Here, the microanatomy indicates two quite different ossification patterns in the nectridean and “microsaurian” lepospondyls. In the former, the early ossification results from an acceleration of the ossification rate of the periosteal region. Similar to the Plagiosauridae, the Nectridea indet. centrum displays a spongy, highly vascularized structure. However, in the “microsaurian” centra, the predominantly periosteal ossification pattern does not seem to be linked to a fast bone deposition rate because in *Microbrachis*, the periosteal region consists of compact, lamellar bone, indicating a rather slow deposition rate (Fig 13A). Thus, it can be assumed that the periosteal ossification process is not faster, but probably starts earlier in vertebral development in the “microsaurian” lepospondyls. An earlier onset of ossification and thereby peramorphosis of skeletal growth in “microsaurian” lepospondyls has also been observed by Olori [79].

As mentioned above, some taxa display a predominance of endochondral ossification. Likewise to the periosteal region, the ossification rate differs among the groups. In stereospondyls, on the one hand, the ossification process is retarded relative to other developmental events. Specifically, in taxa such as *Mastodonsaurus* and *Plagiosuchus*, large amounts of calcified cartilage are preserved in the core of the trabeculae (Figs 5E and 6F). Different histological studies of *Metoposaurus* and *Plagiosuchus* reveal that in individuals of similar ontogenetic stages, calcified cartilage is preserved in the vertebral centra, but not in the long bones [56, 58, 63, 64]. This retention of juvenile features in the adult indicates a paedomorphic condition of the postcranial skeleton [54, 80]. Castantet et al. [52] suggest that this paedomorphic condition is achieved by a slow rate of growth (i.e. neoteny). Conversely, the endochondral growth rate is fast in the seymouriamorph *Discosauriscus*. In the juvenile specimen (DE.KO187-1) that we examined, the trabeculae are strongly remodeled (Fig 8C). The trabeculae consist of parallel-fibered bone and calcified cartilage is absent. This fast rate of endochondral growth has already been observed in the long bones of *Discosauriscus* [55]. Morphological investigations of the vertebral column of *Discosauriscus* showed that the pleurocentra ossify quickly from half-rings to completely ossified rings at a relatively early ontogenetic stage [21].

### Convergent centrum development in miniaturized taxa

Within the rhachitomous temnospondyls, the Amphibamidae display a fascinating vertebral structure [67]: the spool-shaped pleurocentrum has become the main element in the vertebra, whereas the intercentrum is small and crescent-shaped. The outer morphology of the vertebral centrum of *Doleserpeton*, as well as its microstructure (Fig 3), is very similar to the monospondylous pleurocentrum of *Microbrachis* (Fig 13). This is interesting, as both are considered possible close relatives of lissamphibians [7, 23, 81]. In both animals, the slightly thickened periosteal region is composed of nearly avascular, lamellar bone. The endochondral region is compact and large cell lacunae are located around the notochordal canal. As *Microbrachis* (a “microsaurian” lepospondyl) and *Doleserpeton* (an amphibamid temnospondyl) are two phylogenetically remote taxa, this structural similarity must have evolved independently. Moreover, *Microbrachis* and *Doleserpeton* display different lifestyles. The aquatic *Microbrachis* is characterized by a long trunk, a relatively short tail and small limbs [82]. The Lower Permian *Doleserpeton*, in contrast, has well ossified limbs and is found in the highly fossiliferous Fort Sill locality that is interpreted as upland (e.g. [83]) and can therefore be regarded as a terrestrial animal [67, 81]. However, both taxa are miniaturized. Unusual within mostly large temnospondyls, Amphibamidae are small taxa in 5-12cm size range [84]. Carroll et al. [85] assumed that the small body size of lepospondyls is functionally linked to an increase in the ossification rate. The histology of *Microbrachis* sp., however, indicates an earlier onset of periosteal ossification.

A similar pattern is observed in *Doleserpeton* [86]: at an early growth stage, pleurocentra are preserved as thin-walled elements that are ventrally fused to ring-like structures. Thus, the convergent development of the spool-shaped centra is obviously linked to a reduction of body size that, in turn, induces an early ossification process of the periosteal region.

### Vertebral structure and mode of life

Both endochondral and periosteal regions may reveal ecological adaptations. As mentioned above, large amounts of calcified cartilage are preserved in the core of the trabeculae in the aquatic stereospondyls (e.g. *Mastodonsaurus*) (Figs 5 and 6). This retention of denser and highly mineralized calcified cartilage results in an increase of skeletal mass, which functioned as ballast for buoyancy control [87]. The Plagiosauridae are heavily built animals that were mostly interpreted as bottom dwelling aquatic predators [36, 76, 88, 89]. In addition to the retention of calcified cartilage, an increase of the skeletal mass is achieved by a hyperplasy of the periosteal region, i.e. the growth of the periosteal region is increased resulting in a strongly thickened area (Fig 6). This process is called pachyostosis [87, 90]. These results are consistent with previous histological studies of long bones and ribs in plagiosaurids [64, 87, 88] and are also in accordance with their cover of massive osteoderms [91] and a highly ossified neurocranium and palatoquadrate [92]. In contrast, a decrease in skeletal mass characterizes the vertebral centra of the nectrideans. Both the periosteal region and the neural arch have a lightly built spongy structure with numerous intertrabecular spaces and large vascular canals (Fig 12). This osteoporotic-like state fits with the presumed lifestyle of this aquatic group and has already been observed histologically in their dermal bones [93]. Most Permian nectrideans were actively swimming forms with anguilliform locomotion [94]. A slightly different mode of life has been proposed for the nectridean *Diplocaulus* [95]: the boomerang-shaped skull of the aquatic animals functioned as a hydrofoil that enabled them to rise through the water column towards their prey. A decrease of their skeletal mass would certainly facilitate this mode of locomotion.

### The notochordal canal and surrounding tissues

The diameter of the notochordal canal varies within the investigated specimens. In stem-tetrapods and basal temnospondyls, the rhachitomous vertebral centra are formed around a large persisting notochord. During further tetrapod evolution, however, the notochord was increasingly replaced by the vertebral centra and penetrates the centra only as a small canal. As described above, the notochordal canal is surrounded by a distinct bony ring which varies in structure between taxa. For instance, a ring of secondarily remodeled bone trabeculae that are orderly arranged in circular rows surrounds the notochordal canal of *Archeria* (Fig 7A). In comparison, the ring is divided into two parts in *Bystrowiella* with an external ring of compact, primary bone and an internal trabecular structure that underwent resorption followed by deposition of bone that was subsequently remodeled (Fig 11A and 11C). In *Microbrachis* and *Doleserpeton*, the notochordal canal is surrounded by a compact ring with a high amount of large cell lacunae that supposedly housed chondrocytes because these cells are distinctly larger than the osteocyte lacunae and lack canaliculi [96] (Figs 3E and 13C). This implies that the ring was originally formed as a cartilaginous element and was later replaced by bone. Histological and developmental studies in extant fishes and lissamphibians [97–100] have demonstrated that vertebral cartilage can arise within the notochordal sheath (i.e. chordalcentral vertebral formation). As outlined above, Carroll [44] proposed a similar notochordal chondrogenesis in the multipartite vertebral centra of “labyrinthodonts”, i.e. temnospondyls, anthracosaurs, and seymouriamorphs. To the best of our knowledge, our observations in *Microbrachis* and *Doleserpeton* represent the first evidence of a chordacentral vertebral formation in basal tetrapods. The development of the distinct bony ring may have been induced by the notochord itself, implying that both the multipartite and monospondylous vertebral centra formed by chordal and perichordal centra formation. However, considering the limited growth range of the available fossil material, no conclusions can be drawn about sequence, timing, and respective contribution of chordal and perichordal centra formation in vertebral development.

## Conclusions

Both multipartite and monospondylous vertebral centra of stem-tetrapods and basal tetrapods display endochondral and subsequently periosteal ossification. However, the interspecific variability of vertebral development is high: it can either consist of predominantly endochondral ossification (e.g. *Proterogyrinus*) or periosteal ossification (e.g. *Plagiosuchus*). Therefore, the deposition rate of endochondral bone can be slow (e.g. stereospondyls) or fast (e.g. *Discosauriscus*). The same is true for the deposition rate of the periosteal region: *Doleserpeton* displays a slow ossification rate whereas it is fast in *Gerrothorax*. In addition, the initiation of the periosteal ossification process seems to be variable as well. As a result, vertebral development of “microsaurian” lepospondyls started early with predominantly periosteal ossification subsequent to the endochondral ossification process. Therefore, the cartilaginous precursor is not omitted as it was previously assumed [44, 48]. Moreover, vertebral development and structure are influenced by miniaturization (e.g. *Doleserpeton* and *Microbrachis*) and also reflects the individual lifestyle: in the aquatic nectridean *Diplocaulus*, the osteoporotic-like centrum facilitates the floating way of locomotion of these animals. Taken together, the highly variable pattern of vertebral development bears very little phylogenetic signal. This is particularly interesting given the central role that the morphology of vertebral centra has played in the reconstruction of basal tetrapod relationships and specifically the controversy surrounding the competing hypotheses for the origin(s) of lissamphibians [44, 48, 50, 101, 102]. The rather weak phylogenetic signal should not be neglected, if vertebral structure is used to reconstruct phylogenetic trends and relationships. In basal tetrapods, the ossification of the vertebral elements is obviously dependent on numerous biological factors and cannot be reduced to a simple systematic scheme. Interesting examples are the spool-shaped centra of the small temnospondyl *Doleserpeton* and the lepospondyl *Microbrachis* which have both been interpreted as close to the ancestry of lissamphibians. In spite of their distant phylogenetic positions, in both cases centra are characterized by a similar developmental pattern and microstructure. It is nearly impossible to determine which of the two centra may be closer to the developmental origin of the holospondylous lissamphibian vertebrae.
